# Paradoxical Role of Glypican-1 in Prostate Cancer Cell and Tumor Growth

**DOI:** 10.1038/s41598-019-47874-2

**Published:** 2019-08-07

**Authors:** Nhat D. Quach, Sukhneeraj Pal Kaur, Matthew W. Eggert, Lishann Ingram, Deepraj Ghosh, Sheela Sheth, Tamas Nagy, Michelle R. Dawson, Robert D. Arnold, Brian S. Cummings

**Affiliations:** 1 Department of Pharmaceutical and Biomedical Sciences, College of Pharmacy, University of Georgia, Athens, GA USA; 2 Department of Drug Discovery & Development, Auburn University, Auburn, AL USA; 3 Department of Pathology, College of Veterinary Medicine, University of Georgia, Athens, GA USA; 4 Medical College of Georgia, Augusta University, Augusta, GA USA; 5 Department of Molecular Pharmacology, Physiology, & Biotechnology, Brown University, Providence, RI USA; 6 Center for Biomedical Engineering, Brown University, Providence, RI USA; 7 School of Engineering, Brown University, Providence, RI USA; 8 Interdisciplinary Toxicology Program, University of Georgia, Athens, GA USA

**Keywords:** Cancer models, Cancer microenvironment, Cancer microenvironment, Cancer microenvironment, Cancer models

## Abstract

Recent studies suggest that glypican-1 (GPC-1) is a biomarker for prostate cancer, but there are few studies elucidating the role of GPC-1 in prostate cancer progression. We observed high expression of GPC-1 in more aggressive prostate cancer cell lines such as PC-3 and DU-145. While inhibition of GPC-1 expression in PC-3 cells decreased cell growth and migration *in vitro*, it surprisingly increased cell proliferation and migration in DU-145 cells, suggesting that the role of GPC-1 is cell type-dependent. Further, GPC-1 inhibition increased PC-3 tumor size in NCr nude mice xenografts. We hypothesized that the discrepancy between the *in vitro* and *in vivo* data is mediated by stromal cells in the tumor microenvironment. Thus, we tested the effect of tumor conditioned media (TCM) on gene expression in human mesenchymal stem cells and fibroblasts. Treatment of stromal cells with TCM from PC-3 cells transfected with GPC-1 shRNA increased the expression of migration markers, endocrine/paracrine biomolecules, and extracellular matrix components. Additionally, the decreased cell growth in GPC-1 knockdown PC-3 cells was rescued by coculturing with stromal cells. These data demonstrate the paradoxical role that GPC-1 plays in prostate cancer cell growth by interacting with stromal cells and through ECM remodeling and endocrine/paracrine signaling.

## Introduction

Glypicans (GPCs) are heparan sulfate proteoglycans (HSPGs) usually localized at the cellular membrane^1^. Six glypican isoforms have been identified in human cells, referred to as GPC-1 to 6^2,3^. GPCs are reported to regulate cell signaling including cell-to-cell and cell-matrix interactions, Wnt and Sonic hedgehog (Shh) activation, and growth factor binding such as FGF2^1–5^. These signaling pathways affect cellular proliferation, motility and metabolism^6^. More recent studies have suggested roles for GPCs in controlling cell growth in several cancers, including those of the breast, liver, pancreas and prostate^5,7–13^.

While GPCs are known to be expressed in several cancers, their exact role is not understood fully^14^. Inhibition of GPC-1 was reported to impair cellular responses to growth factors such as FGF2, EGF and HGF in PANC-1 (pancreatic carcinoma) cells and decrease tumor formation in nude mice^10^. Further, it was reported that GPC-1 was overexpressed in human breast cancer cells, as compared to non-cancer breast cells, and that inhibition of GPC-1 decreased cell growth and responsiveness to mitogenic growth factors *in vitro*^5^. In contrast, overexpression of GPC-3 in LM3 cells, a mouse mammary tumor cell line, decreased invasiveness and spontaneous lung metastasis compared to controls, prompting the authors to suggest that GPC-3 protected against mammary cancer progression (i.e. it acted as a tumor suppressor). These studies suggest differential roles of GPC isoforms in cancer.

GPCs have also gained attention as possible biomarkers for tumor progression. Increased GPC-1 expression correlated to poor prognosis and decreased survival in patients with pancreatic ductal adenocarcinoma^15^. Higher expression of GPC-1 has also been suggested to predict decreased survival in patients with glioblastoma^16^. GPC-3 has been suggested to be a tumor marker for hepatocellular carcinoma^17,18^, and is reportedly expressed in several other tumors, including ovarian clear-cell carcinoma, neuroblastomas and Wilms’ tumors (see^17^ and references therein). Interestingly, GPC-3 expression appears to be decreased in other tumors, including those from the breast, mesothelioma, ovarian and lung^17,19–21^. This differential expression suggests that the role of GPCs in cancer cell growth is tumor-specific.

GPCs have also been recently suggested to act as biomarkers for prostate cancer^7,12,13^. GPC-5 expression was reported to be lower in prostate cancer tissues isolated from 160 patients, as compared to adjacent normal tissue, and low expression levels correlated to poor prognosis^12^. GPC-1 expression was also higher in neuroendocrine derived prostate cancer cells, DU-145^13^. Further, Suhovskih *et al*. demonstrated that both GPC-1 mRNA and protein expression was detected in low levels in normal prostate tissues, and that these levels increased in prostate tumor tissues. This same study also showed that GPC-1 localization was altered, from the epithelial cells in normal prostate tissues, to the tumor stroma (as opposed to the tumor cells) in the prostate tumors^7^. This alteration suggests that some of the effects of GPC-1 in prostate cancer may be mediated by interaction with the tumor microenvironment. This hypothesis is supported by studies with mouse breast cancer models^8^.

The tumor microenvironment (TME) plays a critical role in cancer. It can either promote^22^, or restrain cancer progression^23–25^, depending on the cancer type and the presence of reactive stromal cells. The TME includes stromal cells (e.g. fibroblasts, immune cells, endothelial cells and mesenchymal stem cells) and extracellular matrix^26^, among other constituents. Interactions between cancer cells and their TME are mediated by cell-cell and cell-matrix interactions^27^, secreted soluble factors^28^ and the stiffness of the environment^29^. As mentioned above, only GPC-5 and GPC-1 have been studied in prostate cancer, and those studies mainly focused on gene expression and localization in human prostatic tissues via *in vitro* experiments using a single cell culture^12,13,30^. The role of GPCs, specifically GPC-1, in prostate cancer cells and stroma signaling exchange has not yet been studied. There is evidence that GPCs are excreted into the extracellular environment^2^, and interact with heparin-binding growth factors such as FGF2 and IGF to facilitate cell growth and migration^5,31^. This prompted us to hypothesize that alteration of GPC-1 expression in cancer cells would affect cancer and stromal responses.

Despite studies suggesting that GPC-1 expression is altered in prostate cancer, and studies suggesting that GPC-1 may be a marker of aggressive prostate cancer, there are little to no studies assessing the functional role of GPC-1 in prostate cancer cell growth or tumorigenesis. This lack of investigation is surprising given that GPCs are suggested to be targets for treatment in liver, breast and pancreatic cancer, and at the least, possible biomarkers. We addressed this gap-in-knowledge by determining the differential expression of GPCs in several prostate cancer cells, which demonstrated increased expression of GPC-1 in more metastatic cells. We assessed the role of GPC-1 in cell growth and tumorigenesis by inhibiting GPC-1 expression and showed a differential response between *in vitro* and *in vivo* tumor models. Assessment of the effect of GPC-1 inhibition on gene expression in stromal cells provide some of the first evidence suggesting that GPC-1 may act a tumor suppressor in prostate cancer via its interaction with the stromal cells.

## Materials and Methods

### Cell culture

PC-3, LNCaP, DU-145, Hs27, and PCS-441-010 cell lines were purchased from ATCC (Manassas, VA) and grown following methods from our previous studies^32^, while human MSCs were acquired from the Texas A&M Health Science Center College of Medicine Institute for Regenerative Medicine. Cell supplements, including antibiotics and primary cell culture media were purchased from ATCC (Manassas, VA). Standard cell culture media were purchased from Corning Inc (Corning, NY). PCS-440-010 (PCS) cells are a primary culture of human non-cancerous prostate cells and were grown in supplemented prostate epithelia cell basal medium according to the manufacture’s recommendations. Human prostate cancer (LNCaP, DU-145 and PC-3) cells were cultured in 10% FBS (Seradigm, Radnor, PA) and 1% penicillin/streptomycin supplemented RPMI-1640, EMEM and F12K, respectively. Human mesenchymal stem cells (hMSC) were cultured in 10% FBS, 1% Pen/Step, and 2.92 mg/mL L-glutamine supplemented alpha-EMEM, while human foreskin fibroblast cells (Hs27) were cultured in DMEM supplemented with 10% FBS and 1% Pen/Step. All cells were incubated in 95% humidity and 5% CO_2_ at 37 °C.

### Quantitative real-time polymerase chain reaction (qRT-PCR)

mRNA was isolated from cells using EZNA® Total RNA Kit I (Promega, Madison, WI) according to the manufacturer’s specifications and as described in our previous publications^32,33^. The quantity and integrity of the RNA was checked using a NanoDrop (Life Science Technology, NY). RNA (1 µg) was converted to cDNA using the iScript cDNA synthesis kit (BioRad, Hercules, CA). cDNA (100 ng) was used for qRT-PCR to analyze the expression of genes listed in Table 1. qRT-PCR was performed using a Bio-Rad iCycler iQ™. Relative expression values were calculated by 2^−ΔΔCt^ using 18S or GAPDH as an internal control^32^. Successfully amplified qRT-PCR cDNA was separated on a 1% agarose gel and extracted using QIAquick Gel Extraction Kits (Qiagen Inc., Germantown, MD). The extracted amplified cDNA was sent to the Georgia Genomics Facility (Athens, GA) for sequence validation. For semi qRT-PCR, only 30 PCR cycles were performed to show differences in gene expression.

**Table 1:** Primers used in this study.

Genes	Primer Sequence	Product Length (bp)
Glypican-1	**F: 5′-**CTTAGTGCTGCTTTGCTTTTCAT**-3′** **R: 5′-**AGGGTTATTATGGGGTGGACTT**-3′**	155
Glypican-2	**F: 5′-**TTAGGAGGGAGTGTGGTTTCC**-3′** **R: 5′-**AAAACTCAACAGAACCCAGGC**-3′**	248
Glypican-3	**F: 5′-**CATGTCTATGCCCAAAGGTAGAG**-3′** **F: 5′-**ATCATCCACATCCAGATCATAGG**-3′**	178
Glypican-4	**F: 5′-**AAGCTGTCTTTGCTTCACGTTAC**-3′** **R: 5′-**TAGCATTTCTTCCAGGTTCACAT**-3′**	200
Glypican-5	**F: 5′-**GGTGTGACTGACAGTTCCCTG**-3′** **R: 5′-**TGCAGATAGTCTGTGGTGTTGAT**-3′**	185
Glypican-6	**F: 5′-**CCCAAGACAGCTACATTTTCAAC**-3′** **R: 5′-**ATACCTCCAAGACAACAGTGCAT**-3′**	195
MMP-9	**F: 5′-**CAACATCACCTATTGGATCC**-3′** **R: 5′-**CGGGTGTAGAGTCTCTCGCT**-3′**	479
Vimentin	**F: 5′-**CTGGATTCACTCCCTCTGGTTG**-3′** **R: 5′-** GGTCATCGTGATGCTGAGAAG**-3′**	112
Zeb-1	**F: 5′-**TTCAAACCCATAGTGGTTGCT**-3′** **R: 5′-** TGGGAGATACCAAACCAACTG**-3′**	268
Zeb-2	**F: 5′-**GCACAAGACTACATGTCAGGCC**-3′** **R: 5′-**CACACTGATAGGGCTTCTCGC**-3′**	152
N-Cadherin	**F: 5′-**CCATCAAGCCTGTGGGAATC**-3′** **R: 5′-**CTGTGGGGTCATTGTCAGCC**-3′**	147
E-Cadherin	**F: 5′-**CTCGTAACGACGTTGCACC**-3′** **R: 5′-**CTGTGGGGTCAGTATCAGCC**-3′**	123
CXCR4	**F: 5′-**ACGTCAGTGAGGCAGATG**-3′** **R: 5′-**GATGACTGTGGTCTTGAG**-3′**	202
ROCK1	**F: 5′-**TTACTGACAGGGAAGTGAGGTT**-3′** **R: 5′-**AGGTAGTTGATTGCCAACGAAA**-3′**	230
ROCK2	**F: 5′-**AACAGGCATGGTACATTGTGAT**-3′** **R: 5′-**GGAAAACACCTACAGACCACC**-3′**	128
Acta2	**F: 5′-**ACCCAGCACCATGAAGATCA**-3′** **R: 5′-**TTTGCGGTGGACAATGGAAG**-3′**	157
CDC42	**F: 5′-**CATTTGTTTGCCATTTGCTG**-3′** **R: 5′-**ACCACCCCTCGTATTTCCTC**-3′**	240
Pak-1	**F: 5′-**CGTGGCTACATCTCCCATTT**-3′** **R: 5′-**GGATCGCCCACACTCACTAT**-3′**	150
Rac-1	**F: 5′-**AACCAATGCATTTCCTGGAG**-3′** **R: 5′-**TGTTTGCGGATAGGATAGGG**-3′**	154
RhoA	**F: 5′-**TATCGAGGTGGATGGAAAGC**-3′** **R: 5′-**TTCTGGGGTCCACTTTTCTG**-3′**	172
PLA2G2A	**F: 5′-**TGTGTGAGTGTGATAAGGCTG**-3′** **R: 5′-**GGAGGGTATGAGAGAGGGAAA**-3′**	175
SDF-1	**F: 5′-**GTGTCACTGGCGACACGTAG**-3′** **R: 5′-**TCCCATCCCACAGAGAGAAG**-3′**	263
TGFb3	**F: 5′-**GCGGAGCACAACGAACTGG**-3′** **R: 5′-**ATCAAAGGACAGCCACTCGG**-3′**	267
IGF2	**F: 5′-**GTGACCAGCAAGGCACAAATC**-3′** **R: 5′-**CACCAAGTAGGCACCACTAAG**-3′**	245
Col5a1	**F: 5′-**TACAACGAGCAGGGTATCCAG**-3′** **R: 5′-**ACTTGCCATCTGACAGGTTGA**-3′**	136
FN1	**F: 5′-**GAGAATAAGCTGTACCATCGCAA**-3′** **R: 5′-**CGACCACATAGGAAGTCCCAG**-3′**	200
HAS1	**F: 5′-**TCAAGGCGCTCGGAGATTC**-3′** **R: 5′-**CTACCCAGTATCGCAGGCT**-3′**	195
GAPDH	**F: 5′-** AGCCACATCGCTCAGACAC **-3′** **R: 5′-**TGGAAGATGGTGATGGGATT**-3′**	221
18S	**F: 5′-**GTAACCCGTTGAACCCCATT**-3′** **R: 5′-**CCATCCAATCGGTAGTAGCG**-3′**	151

### Immunoblot analysis

Proteins from different cell lines and tumor samples were collected in RIPA buffer supplemented with 1% (v/v) protease inhibitor cocktail (Sigma, St. Louis, MO). The protein concentrations were determined using the BCA assay and 40 µg of total protein was separated on 4%/12% stacked SDS-PAGE gels, and transferred to nitrocellulose membranes, as described in our previous publications^32,33^. The membranes were blocked in 5% (w/v) milk powder TBS-T for 2 hours and exposed to antibodies. Mouse monoclonal anti human GPC-1 (sc-101827), MMP-9 (sc-21733) antibodies and rabbit polyclonal anti N-cadherin (N-Cad, sc-7939) and E-cadherin (E-Cad, sc-7870) were purchased from Santa Cruz Biotechnology (Santa Cruz, CA) and incubated with the membrane at a 1:500 dilution in TBS-T for at least 2 hours or overnight at 4 °C, while GAPDH (sc-32233), which was used as a loading control, was used at a 1:200 dilution in TBS-T and incubated for 1 hour at room temperature^32,33^. Membranes were incubated with a relevant peroxidase conjugate secondary antibody (Promega, Madison, WI) at a 1:2500 dilution for 2 hours. Bands were visualized using SuperSignal Chemiluminescent substrate (Thermo Scientific, Waltham, MA) and intensities visualized and quantified using an Alpha Innotech FluorChem™ HD2 system (ProteinSimple, Santa Clara, CA).

### Plasmid transfection and lentiviral transduction

293-Ta cells were seeded at 100,000 cells/ml in 10-cm cell culture dishes and incubated for 24 hours before being transfected with 8 µg of control or GPC-1 shRNA plasmids (CTGCTCGCTCCTTTGTGCA) (Genecopoeia, Rockville, MD), along with packaging plasmids using plasmid transfection reagent (Polyplus-transfection Inc., New York, NY) in serum-free DMEM according to the manufactures recommendation. Control and GPC-1 shRNA plasmids contained GFP as a reporter gene to ensure the success of transfection. As previously described^32^, cells were incubated overnight and later incubated in fresh serum-free DMEM for an additional 24 hours to produce lentivirus. Lentiviral containing media were collected, filtered through a 0.45 µm filter, and concentrated by centrifugation. PC-3 cells were infected with various titers of GPC-1 shRNA lentiviral vectors in media containing 8 µg/ml of polybrene, while the control cells were transfected with a scrambled plasmid containing virus. The medium was aspirated after 24 hours and replaced with growth media and incubated overnight. Clonal selection was performed the following day using 1 µg/ml of puromycin. Immunoblot analysis was performed on scrambled shRNA and GPC-1 shRNA PC-3 cells after transfection to verify knockdown.

Additional inhibition of GPC-1 was induced using lentiviral particles containing GPC-1 targeting shRNA (TRCN0000122911) (Sigma, St. Louis, MO) while scrambled shRNA containing lentiviral particles were used as control (SHC002V). Sequences of these shRNAs can be found at the manufacturer’s website. Lentiviral transduction of PC-3 and DU-145 cells was performed according to the manufacturer’s protocol. Briefly, 1.02 × 10^4^ cells were seeded in a 96-well plate. All treatments were done in duplicate. After overnight incubation at 37 °C in 5% CO_2_, each well was treated with 50 µL complete media (PC-3 and DU-145 RPMI) containing 16 µg/mL polybrene. The volume of lentiviral particles with multiplicity of infection (MOI) of 5 was calculated for each construct per well and mixed in 50 µL media and added to appropriate wells. After 24 hours, media containing lentiviral particles was aspirated and replaced with 100 µL of fresh media. After 24 hours, cells were selected using puromycin (PC-3: 2 µg/mL, DU-145: 0.5 µg/mL), followed by expansion of each clone. qRT-PCR analysis and western blot analysis was performed on control and GPC-1 shRNA cells to confirm the knockdown.

### Measurement of crystal violet staining

Cells were seeded in 24-well plates (1,000 cells per well) and incubated for the desired times. These conditions were based on our previous study using some of these same cells^32^. Cells were fixed with 10% formalin (Sigma, St. Louis, MO) for 10 minutes, and stained with 1% (w/v) crystal violet for 15 minutes followed by at least 3 washes with water. The crystal violet stain was visualized under phase contrast microscopy or was dissolved in 2% SDS in PBS and the absorbance was determined at 540 nm with a FLUOstar OPTIMA (BMG Lab technologies, Inc., Durham, NC) plate reader. Absorbance readings were normalized to scrambled PC-3 as controls for each time point.

### Scratch wound assay

Cells were seeded in 6 well plates at 400,000 cells/ml and incubated for at least 24 hours or until reaching confluence. 1 mL pipette tips were used to create an uniform scratched area on the cell layer. The ability of cells to close the wounded area were measured after 48 or 72 hours (depending on cell type) using phase contrast or fluorescent microscopy on an X71 inverted epifluorescent microscope with a Peltier element-cooled 12.8MP DP72CCD camera and CellSens software (Olympus). Cell migration rate was evaluated by the percentage of gap closure^34^.

### Invasion assay

8 µm-pored sized trans-well chambers (Corning Corp, Corning, NY) were used to compare the migration ability of cells. A thin layer of 200 µg/ml Growth Factor Reduced (GFR) Matrigel^TM^ (BD, San Jose, CA) was coated on the top chamber to provide an additional barrier for cell migration. A total of 100,000 cells were seeded in 200 µl serum-free DMEM onto the top chamber while 10% FBS supplemented DMEM was applied to the bottom chamber. Cells were incubated for 24 hours prior to analysis of migration. Cells were fixed in formalin, and cells on the top chamber were removed gently using a cotton swab. Cells on the other side of the membrane were visualized using an X71 inverted epifluorescent microscope with a Peltier element-cooled 12.8MP DP72CCD camera and CellSens software (Olympus). Five random areas were captured and counted for the number of cells in each area.

### Matrigel™ colony formation assays

GFR Matrigel™ (Corning Inc, Corning, NY) were used to determine the role of extracellular matrix in tumor formation from a single cell imbedded in a semi-3D architecture. In brief, approximately 400 of control or GPC-1 knockdown PC-3 cells were mixed in ice-cold 125 µg/ml GFR Matrigel™ and seeded into 96-well plates with a volume of 80 µL per well. The GFR Matrigel™ and cell mixtures were incubated at room temperature for 30 mins. 50 µL of normal growth media was then added to the top of the solidified gel. Media were changed every three days and cell colonies were evaluated based on the diameter of the spheroid (reported in pixel units measured in ImageJ) after 10 days of culture by taking 5 random images per condition in triplicates and repeated with n of 3 independent experiments. Total of 45 spheroids were analyzed per condition.

### Adhesion assay

Cell adhesion was assessed in the presence and absence of pre-seeded fibroblasts in 96-well clear bottom black plates (Corning Inc, Corning, NY). Since control and GPC-1 cells expressed GFP as a reporter gene in the transfected plasmids, we used GFP as a readout that correlated to cell number in this assay. In short, cells were seeded at 40,000 cells per well in serum and phenol dye free DMEM and incubated for 4 hours. Cells were then washed 3 times with serum and phenol-free DMEM before measuring the number of adhered cells by fluorescent spectroscopy at 488/512 nm (excitation/emission wavelength) using a SpectraMax® M5 (Molecular Devices, Sunnyvale, CA).

### Collection of tumor conditioned media (TCM)

Scrambled and GPC-1 knockdown PC-3 cells were cultured to confluence in 10-cm dishes in normal growth media, then washed with PBS, and incubated in 8 ml serum-free DMEM for 24 hours. TCM was collected in a single batch, centrifuged at 1000 × g and filtered to eliminate cells and debris. TCM was aliquoted and stored at −80 °C for future use.

### Coculture of cancer and stromal cells

Approximately 100 scrambled or GPC-1 shRNA PC-3 cells, which had GFP fluorescence as a reporter gene, were co-cultured with 10,000 hMSCs or Hs27 cells in 96-well clear bottom black plates (Corning Inc, Corning, NY) and cultured in 10% FBS, 1% Pen/Step, and 2.92 mg/mL L-glutamine supplemented DMEM for 7 days. Scrambled and GPC-1 shRNA PC-3 cell colonies were distinguished from stromal cells by their GFP expression in the culture plates and cell growth was monitored over 7 days. Cell morphology was assessed using an X71 inverted epifluorescent microscope with a Peltier element-cooled 12.8MP DP72CCD camera and CellSens software (Olympus, Pittsburgh, PA). Cell density was determined by measuring GFP fluorescent intensity using a SpectraMax® M5 spectrophotometer (Molecular Devices, Sunnyvale, CA) at 488/512 nm in phenol-free DMEM.

### Gelatin zymography

hMSCs and Hs27 at ~90% confluence were cultured in serum-free DMEM or TCM collected from control and GPC-1 KD PC-3 cells for 24 hours in 6-well plates. The cultured media was then centrifuged, and 20 µl of the media was mixed with 5x loading buffer (250 mM Tris·HCl, pH 6.8, 10% SDS, 30% (v/v) glycerol, and 0.05% (w/v) bromophenol blue) and loaded onto 10% polyacrylamide gel embedded with 0.1% w/v gelatin for protein separation. Gels were rinsed with water and subjected to two sequential incubations with renaturing buffer (1% Triton X-100, 0.02% w/v Brij-35, 0.02% NaN_3_, 50 mM Tris base at pH 7.5, 5 mM CaCl_2_, and 200 mM NaCl) for 30 minutes with agitation. Gels were then rinsed with water and preincubated in developing buffer (0.02% w/v Brij-35, 0.02% NaN_3_, 50 mM Tris base at pH 7.5, 5 mM CaCl_2_, and 200 mM NaCl) for 60 minutes. After that, gels were placed in fresh developing buffer and incubated for at least 16 hours at 37 °C. Gels were stained in 0.25% w/v Coomassie blue diluted in methanol: acetic acid: water mixture (1:8:1 ratio) followed by de-staining with the methanol: acetic acid: water mixture. Gels were then scanned using an HP ScanJet.

### *In Vivo* Xenograft mouse model

All animal handling and experiments were performed under a protocol approved by the Institutional Animal Care and Use Committee (IACUC) at Auburn University and in accordance with the US. Public Health Service (PHS) Policy on Humane Care and Use of Laboratory Animals, updated, 2015. Xenografts of GPC-1 knockdown (GPC-1 shRNA) and control PC-3 cells were established subcutaneously in the left flank of NCr nude, 6–8 week-old male mice (Taconic Biosciences Inc., Albany, NY) by injecting 200 µl of 1 × 10^6^ suspended cells in ice-cold 5 mg/ml Matrigel® (1:1) mixture. During tumor implantation, mice were supplied with 1–3% isoflurane gas (Henry-Schein, Melville, NY) mixed with oxygen to induce and maintain anesthesia. Implants were allowed to set for 5–10 minutes before allowing the mice to recover from anesthesia. Tumors of control and GPC-1 knockdown cells were performed in two independent experiments using 11 mice per cell type to evaluate the role of GPC-1 in tumor growth. Vitals and tumor growth were assessed weekly by recording mouse weight and by obtaining digital caliper measurements of observable length and width dimensions of the subcutaneous tumor to determine volume. Volumes were calculated assuming the volume of an ellipsoid: *Caliper Tumor Volume* = *(π/6)(larger diameter)(smaller diameter*)^2^, as we have described previously^35^. At the end of the experiment, mice were euthanized by carbon dioxide asphyxiation. Tumor tissues were collected rapidly and either flash-frozen in liquid nitrogen for immunoblot analysis or fixed in 10% buffered formalin for immunohistochemistry.

### Hematoxylin and Eosin (H&E) staining

Tumor tissues were fixed in 10% buffered formalin overnight, embedded in paraffin wax and sectioned into 10-μm thick specimens. Paraffin sections were deparaffinized, rehydrated, and subjected to routine hematoxylin and eosin staining histological processes. Stained sections were mounted with Fluoromount (Sigma Aldrich, St. Louis, MO) and visualized using a Nikon AZ100 microscope (Tokyo, Japan) as previously reported by us^33^. H&E slides were analyzed in a blinded fashion at the Histology Laboratory at the University of Georgia School of Veterinary Medicine.

### Immunohistochemistry and fluorescence staining

Serial 10-μm-thick sections were deparaffinized in xylene, rehydrated in a 100–70% descending series of ethanol, immersed in citrate buffer (pH 6.0) and incubated in a humidified chamber to facilitate antigen retrieval.

For double fluorescence immunohistochemistry, tumor paraffin-embedded sections were incubated overnight at 4 °C with a monoclonal rabbit anti-E-cadherin (Cell Signaling Technology, Beverly, MA) and a monoclonal mouse anti-N-cadherin (D-4) antibody from (Santa Cruz Biotechnology, Santa Cruz, CA) at 1:250 and 1:50 dilutions in 2% BSA-0.1% Triton-X PBS, respectively. Three PBS washes were performed before incubating with secondary antibodies (Alexa Fluor 488 goat anti-rabbit IgG and Alexa Fluor 594 goat anti-mouse IgG (Invitrogen, Merelbeke, Belgium)) at a 1:1000 dilution for 1 hour at room temperature. Slides were rinsed with PBS and mounted with Vectashield mounting medium with DAPI (Labconsult, Brussels, Belgium).

For Ki-67 staining, antigen retrieved slides were placed in 3% H_2_O_2_/ultra-pure water for 20–30 minutes at room temperature to block endogenous peroxidase activity. Subsequently, sections were processed for immunohistochemistry by using the manufacturer’s protocol for the Vectastain Universal Elite ABC HRP Kit (Vector Laboratories, Burlingame, CA). The rabbit anti-Ki-67 primary antibody (Novus Biologicals, Littleton, CO) was used at a 1:400 dilution in 2% BSA-0.1% Triton-X PBS. Then, slides were incubated in the appropriate secondary antibody (Novus Biologicals, Littleton, CO) and later counterstained with hematoxylin. Slides were prepared as previously described by us^33^, and were rinsed with PBS and mounted Fluoromount (Sigma Aldrich, St. Louis, MO). Quantification and image analysis was subsequently performed using the Image-J based software package FIJI^36^. Using the ‘colour deconvolution’ with ‘H DAB’ settings, DAB and Gill’s hematoxylin staining were deconvoluted, inverted, and converted into 8-bit gray scale images. Regions of interest (ROI) were defined to ensure uniform quantitative comparisons across sections. Relative optical density (O.D.) was calculated based on DAB staining values where mean intensity was calculated using ‘Measure’ function. A reciprocal intensity was derived based on the maximum intensity of an 8-bit image (250) and log transformed to yield an O.D. directly proportional to the amount of chromogen present. Tissue staining was visualized using a Nikon AZ100 microscope (Tokyo, Japan).

### Statistical analysis

All experiments were completed at least three times. Results are shown as the average of all replicates ± SEM. Data were analyzed using a student t-test’s or a two-way ANOVA followed by Dunnett’s post-hoc test as indicated in the figure legends if applicable, and differences with a *p*-value < 0.05 were considered significant.

TCGA Prostate Cancer (PRAD) Kaplan Meier (KM) survival plots were constructed based on expression of GPC-1 in 496 primary prostate tumor samples using UCSC Xena Cancer Browser Database (http://xena.ucsc.edu/). A log-rank test was automatically used as a default by Xena Browser to generate the test statistics (*χ*2) and p-value (*χ*2 distribution).

## Results

### Expression of GPC isoforms in prostate cancer cells

**Figure 1:**
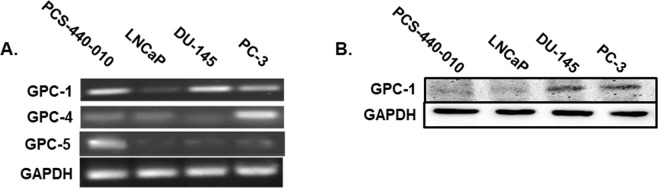
Expression of GPC-1 in Prostate Cell Lines. (**A**) Expression of GPC-1, 4, 5 mRNA in PCS-440-010 (primary non-cancer) and LNCaP, DU-145 and PC-3 (human prostate cancer) cells. (**B**) GPC-1 protein expression in PSC-440-010, LNCAP, DU-145 and PC-3 cells. Data are representative of at least 3 independent cell passages.

Few studies exist determining the expression of different GPCs in prostate cancer cells in comparison with normal prostate cells. We performed a comparative mRNA analysis of primary prostate epithelial cells (PCS-440-010) against prostate cancer cells (LNCaP, DU-145 and PC-3) using semi qRT-PCR. Expression of GPC-1, -4 and -5 mRNA was detected in both primary prostate epithelial cells and cancer cells (Fig. 1A); however, GPC-2, 3, and 6 mRNA were expressed at lower level and were not detected using semi qRT-PCR in which the polymerase reaction was terminated at cycle 30^th^. GPC-1 mRNA was highly expressed in primary prostate epithelial cells and metastatic prostate cancer cells (DU-145 and PC-3). GPC-5 mRNA was more abundant in primary prostate epithelial cells than the cancer cells, and these levels tended to decrease in the more metastatic prostate cancer cell lines such as DU-145 and PC-3 cells, as compared to a less metastatic cell line (LNCaP). GPC-4 mRNA was relatively higher in PC-3 cells, as compared other cells. Interestingly, protein expression of GPC-1 was higher in DU-145 and PC-3 cells, as compared to either PCS-440-010 or LNCaP cells (Fig. 1B).

### Effect of GPC-1 inhibition on cell proliferation of prostate cancer cells

**Figure 2:**
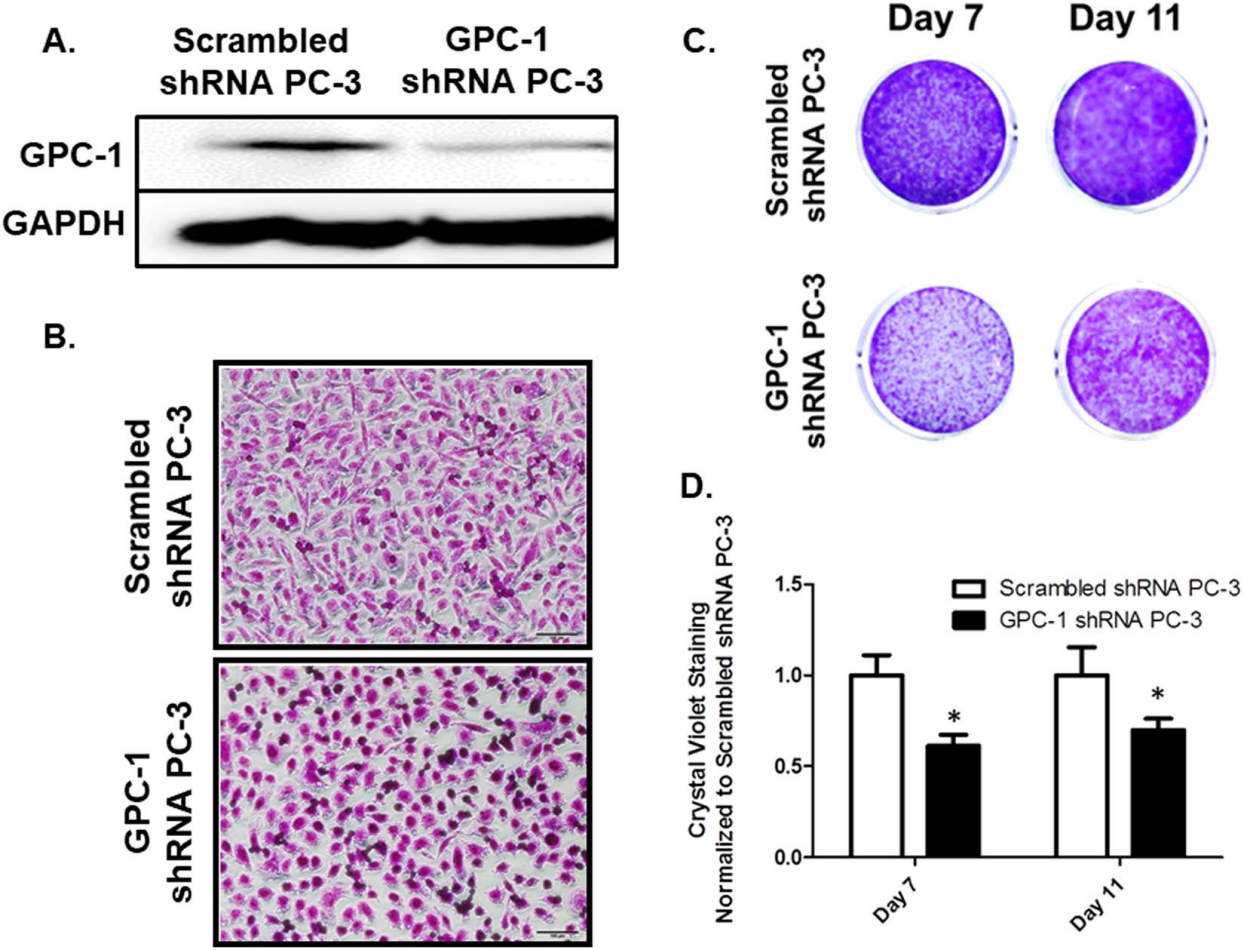
Effect of GPC-1 Inhibition on Prostate Cancer Cell Growth and Morphology. (**A**) Effect of GPC-1 shRNA (Genecopoeia, Rockville, MD) on expression of GPC-1 in PC-3 cells. (**B**) Effect of GPC-1 inhibition on cell morphology visualized by crystal violet staining at high seeding density. (**C**,**D**) Changes in cell growth in GPC-1 knockdown PC-3 cells visualized by crystal violet staining after 7 and 11 days of culture (**C**) followed by quantification (**D**) Data in (**A**,**C**,**D**) are representative of at least 3 separate experiments with at least 3 independent cell passages. Data in **D** are presented as the mean ± the S.E.M. of at least 3 (n = 3) separate passages. *Indicates a significant difference (*p* < 0.05) as compared to control as determined using a Student t-test.

We focused our studies on GPC-1 based on previous literature reports that GPC-1 could be a biomarker for aggressive prostate cancer^13^. In addition, we observed high protein expression levels of GPC-1 in metastatic cancer cell lines such as DU-145 and PC-3. PC-3 cells were used as a cell model because they are derived form a bone-marrow metastatic prostate cancer origin, and bone metastases leads to a poor prognosis in prostate cancer patients^37^. Stable transfection of PC-3 cells with shRNA targeting GPC-1 decreased GPC-1 protein expression, as compared to cells transfected with scrambled shRNA as a control (Fig. 2A). Inhibition of GPC-1 expression altered cell morphology to a less mesenchymal-like shape (Fig. 2B). Additionally, inhibition of GPC-1 expression decreased crystal violet staining after both 7 and 11 days of culture (Fig. 2C,D). We further inhibited expression of GPC-1 in DU-145 cells (Supplemental Fig. 1A). We were unable to create a stable DU-145 knockdown cells line for DU-145 because the cells did not stably maintain the knockdown genotype over few cell passages. We hypothesized that GPC-1 could affect the ability of DU-145 cells to adhere to our culture dish in our routine cell culture procedure, resulting in the loss of knockdown genotype over cell passages. A proper GPC-1 inhibition method will be required for this cell lines to accurately study the role of GPC-1 in this cell line. Nevertheless, transient inhibition of GPC-1 in DU-145 cells resulted in a more mesenchymal cell morphology (Supplemental Fig. 1B) and an increase of cell proliferation and migration (Supplemental Fig. 1C,D). These data suggest that the role of GPC-1 in cancer cells may be cell-type dependent. Further, analysis of differential expression of other GPC isoforms showed that inhibition of GPC-1 resulted in alteration of other GPC isoforms in cell-dependent manner (Supplemental Fig. 2). The expression of GPC-2, -3, and -6 were detected in PC-3 cells; however, the amplification was later than cycle 30^th^, suggesting the expression is very low in the cells. Notably, mRNA levels of GPC-5 and -6 were either decreased or unchanged in PC-3 cells in which GPC-1 was inhibited, while increased in DU-145 cells. GPC-3 expression was decreased in PC-3 cells transfected with GPC-1 shRNA. We focused our remaining studies on PC-3 cells due the relevance of this cell line to bone-metastasized prostate cancer in the clinic as well as the ability to generate multiple stable knockdown cell lines.

### Effect of GPC-1 inhibition on prostate cancer cell migration, adhesion and spheroid formation

**Figure 3:**
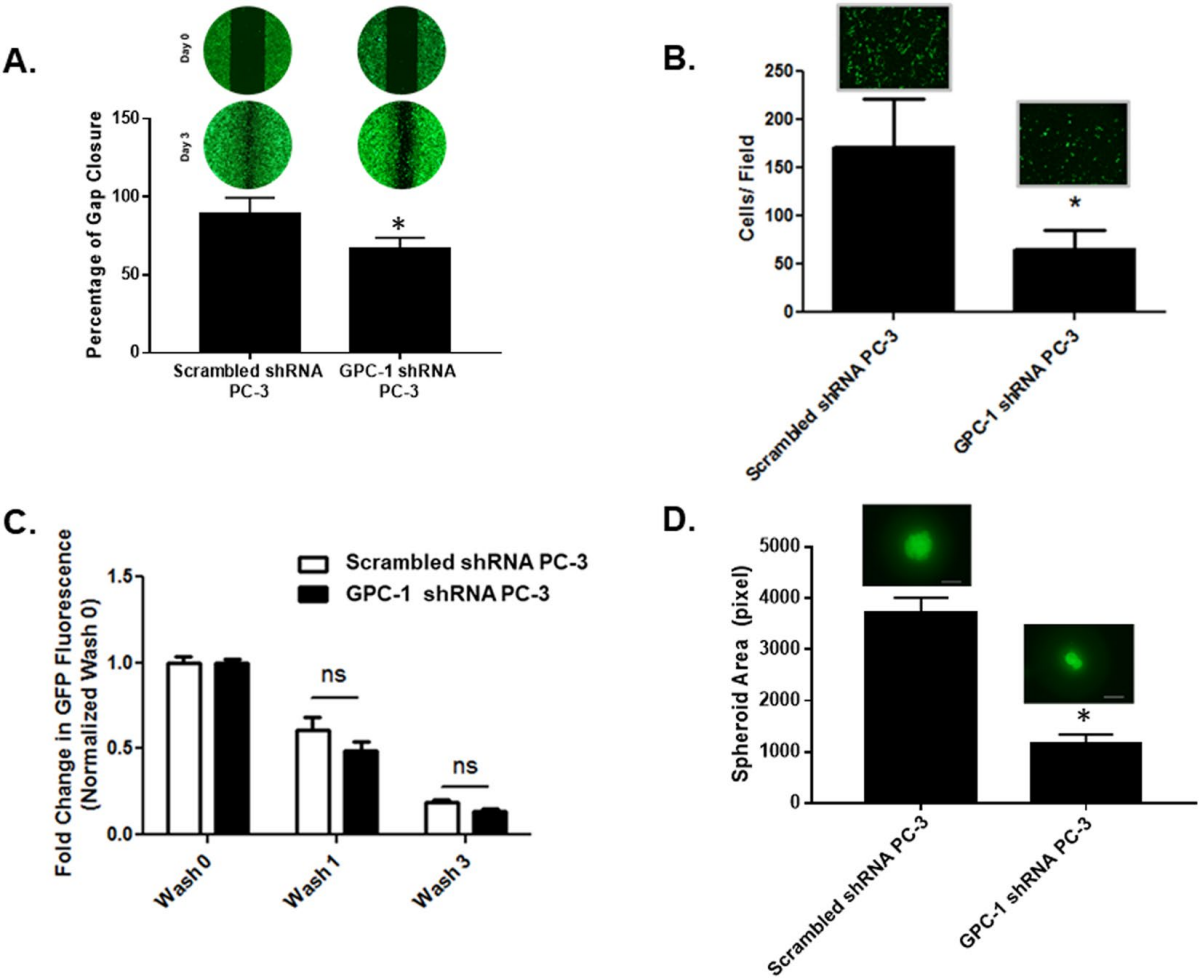
Effect of GPC-1 Inhibition on Prostate Cancer Cell Migration, Adhesion and Spheroid Formation. Scratch (**A**) and trans-well assays (**B**) were used to determine the effect of GPC-1 inhibition on PC-3 cell migration. Adhesion assays (**C**) were conducted to determine cell adhesion on a plastic cell culture dish after 4 hours of incubation. Spheroid formation (**D**) was tested using a Matrigel™ colony assay. The scale bar in (**D**) indicates 200 µm. Data in (**A**,**D**) are representative of at least 3 (n = 3) experiments performed on 3 independent passages. Data in (**B**,**C**) are presented as the mean ± the S.E.M. of at least 3 independent passages. *Indicates a significant difference (*p* < 0.05) as compared to control as determined using a Student t-test. The ns in **C** indicates a non-significant difference as compared to the control.

**Figure 4:**
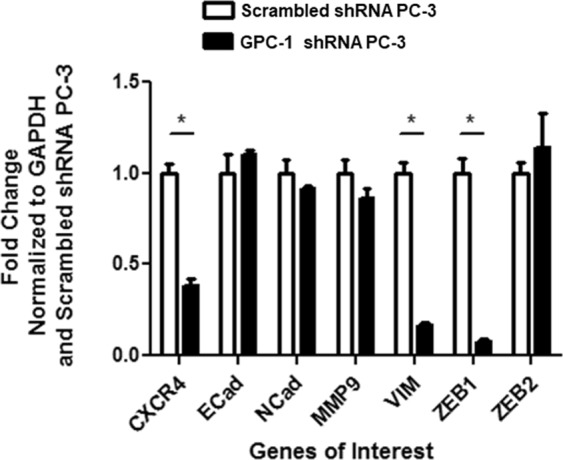
Effect of GPC-1 Inhibition on the Expression of Select Cancer-related Genes. GPC-1 expression was inhibited in PC-3 cells using shRNA (Genecopoeia, Rockville, MD) and the effect of inhibition on the mRNA levels of genes related to cancer cell progression was determined using qPCR. Data are presented as the mean ± the S.E.M. of at least 3 separate passages. *Indicates a significant difference (*p* < 0.05) as compared to control as determined using a student t-test.

We assessed the effect of GPC-1 inhibition on cell migration using both scratch and trans-well assays (Fig. 3). These assays demonstrated that GPC-1 knockdown cells migrated slower than control PC-3 cells, based on a decrease in the apparent wound gap closing after 3 days (α. 3A), and the impairment in the migration of cells through the 8 µm pores of the trans-well membrane after 24 hours (Fig. 3B). Additionally, there was no significant difference in cellular adhesion between control and GPC-1 knockdown cells after multiple washes with DMEM (Fig. 3C). In contrast, inhibition of GPC-1 expression decreased spheroid formation after 10 days in a single cell culture in GFR Matrigel™ matrix as compared to control cells. Furthermore, inhibition of GPC-1 decreased several markers of prostate cancer aggression, such as MMP-9, E-Cadherin (E-Cad), N-Cadherin (N-Cad), CXCR4, vimentin (VIM), Zeb1 and Zeb2^38–40^, as determined using qRT-PCR (Fig. 4).

We verified these data by generating additional GPC-1 knockdown cell lines using shRNA with differential sequences (see methods). Similar to data reported in Figs 2 and 3, inhibition of GPC-1 using this additional shRNA altered cell PC-3 cell morphology, and decreased cell proliferation and cell migration (Supplemental Fig. 3). Interestingly, inhibition of GPC-1 resulted in aslight increase in the expression of GPC-5 in PC-3 cells, but the increase is not significant with a p value of 0.3093. Meanwhile, no changes were detected in GPC-4 mRNA level (Supplemental Fig. 2). Collectively, these data support the hypothesis that GPC-1 mediates the growth and migration of prostate cancer cells *in vitro*.

### Effect of GPC-1 inhibition on prostate cancer cell growth *in vivo*

**Figure 5:**
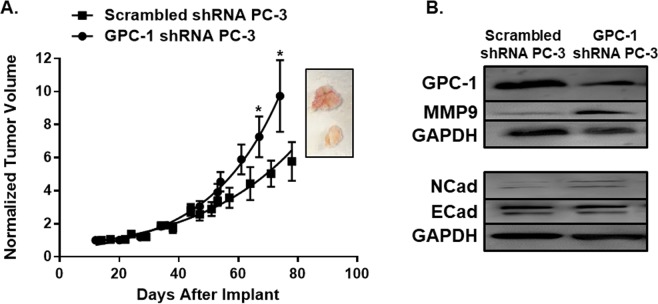
Effect of GPC-1 Inhibition on Tumor Growth in Using NCr Nude Mice Xenografts. **(A**) PC-3 cells transfected with either scrambled shRNA or shRNA against GPC-3 were used to establish tumors whose growth were tracked based on tumor volume for 80 days. (**B**) The effect of shRNA against GPC-1 on the expression of GPC-1 and other proteins were determined by immunoblot analysis. Data in **A** are presented as the mean ± the S.E.M. of 6-11 (n = 6–11) difference mice conducted in at least two independent cohorts. Data in **B** are representative of at least 3 animals. *Indicates a significant difference (*p* < 0.05) as compared to control as determined using a Student t-test.

We determined the effect of GPC-1 inhibition on tumor growth by subcutaneously implanting PC-3 cells transfected with either GPC-1 shRNA or scrambled shRNA into 6–8 week old male athymic nude (NCr, nu/nu) mice. The engrafted tumors were allowed to form and grow for ~11 weeks at which time the study was stopped due to predetermined limitations in tumor size. Quite surprisingly, and in total contrast to the *in vitro* studies, tumors formed from GPC-1 shRNA transfected PC-3 cells grew to almost twice the size of PC-3 cells expressing the scrambled shRNA (Fig. 5A). Immunoblot analysis demonstrated that GPC-1 expression was still decreased in GPC-1 knockdown tumors, as compared to scrambled controls. Analysis of the Hematoxylin and Eosin (H&E) staining showed varying degrees of vacuolization comparable with lipid vacuoles. The degree of vacuolization did not appear to differ between groups. In contrast, the increase in tumor growth correlated to decreases in necrosis in tumors formed from GPC-1 shRNA (Supplemental Fig. 4). There was no difference in KI-67 staining between the two tumors (Supplemental Fig. 5).

Numerous studies suggest that GPC-1 mediates interaction between cancer cells and the tumor microenvironment (TME)^2,41–43^. The studies also suggest that GPC-1 can alter the behavior of the TME^8,10,15,44–47^. However, these studies mostly focused on other cancer types as opposed to prostate cancer. Our observation of the paradoxical behaviors in prostate tumor growth between *in vitro* and *in vivo* models led us to hypothesize that inhibition of GPC-1 expression in prostate cancer cells remodeled the TME and subsequently affected tumor growth by mediating cancer-stromal cell interactions. The *in vitro* culture models employed above, in which tumor-surrounding cells are absent, would not be able to fully assess these interactions. To explain the differences between these two different models, we examined the expression of MMP-9, E-Cad and N-Cad, which are known to mediate TME^38–40,48^. In agreement with the increase in tumor growth in mice injected with GPC-1 shRNA PC-3 cells, MMP-9 protein expression was higher in these tumors as compared to controls (Fig. 5B). Further, while E-Cad protein levels remained similar between GPC-1 shRNA PC-3 tumors and scrambled controls, N-Cad levels were increased (Fig. 5B). The increased expression of N-Cad in GPC-1 shRNA PC-3 cell derived tumors was confirmed using immunofluorescent staining (Supplemental Fig. 6).

### Effect of GPC-1 inhibition on gene expression in stromal cells and prostate cancer cell growth in a coculture model

**Figure 6:**
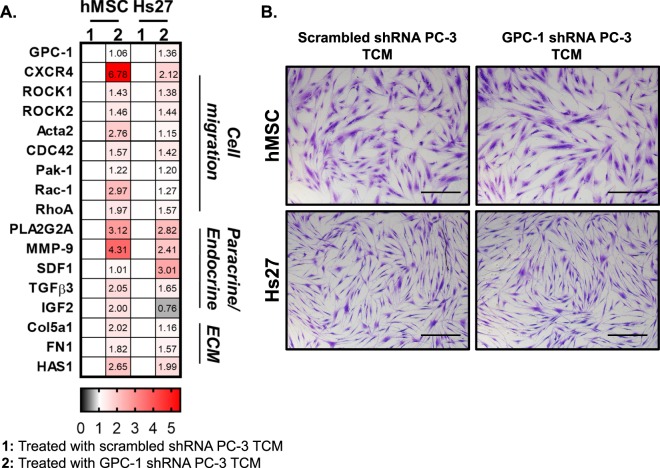
Effect of GPC-1 Inhibition on Stromal Cell Gene Expression. (**A**) hMSC or Hs27 cells were exposed to TCM was isolated from either control PC-3 cells (scrambled shRNA) or from PC-3 cells transfected with GPC-1 shRNA for 24 hours after which the expression of the indicated genes was determined by qRT-PCR. Data are represented as a heatmap illustrated the fold-change in mRNA expression of hMSC and Hs27 treated with TCM using qRT-PCR. Fold change was calculated based on normalization with cells treated with scrambled shRNA PC-3 TCM. The select genes were categorized into groups that are known to mediate cell migration, paracrine/endocrine signaling and ECM. (**B**) Morphology of stromal cells under TCM was visualized after crystal violet staining. The scale bar in (**B**) indicated 50 µm. Stromal cells. Data in **A** are presented as mean of at least 3 independent experiments.

To investigate the effect GPC-1 inhibition on gene expression in the TME we tested the effect of tumor condition media isolated from PC-3 cells on gene expression in human stromal cell lines such as mesenchymal stem cells (hMSCs) and fibroblasts (Hs27). These stromal cells were chosen because they have been shown to migrate to neoplastic niches and to orchestrate changes in the TME^28,49–52^. Thus, we collected tumor soluble factors secreted by GPC-1 shRNA and scrambled shRNA PC-3 cells into serum media after 24 hours of incubation. The tumor conditioned media (TCM) were applied to hMSC and Hs27 cells and incubated for additional 24 hours before MMP gelatin zymography analysis for analysis of MMP activity (Supplemental Fig. 7) and gene expression as analyzed using qRT-PCR (Fig. 6A). As hypothesized, MMP-9 activity was increased in both Hs27 and hMSC cells exposed to TCM from GPC-1 shRNA PC-3 cells, as compared to control TCM or serum-free DMEM. In addition, MMP-2 activity was detected in hMSC treated cells but not in Hs27; however, there was no difference in MMP-2 activity between hMSC treated with GPC-1 KD TCM and controls (Supplemental Fig. 7).

Many studies have shown the involvement of stromal cells in orchestrating the TME to support or inhibit cancer progression^53–57^. We assessed the role of GPC-1 in cancer-stromal interactions by examining mRNA expression of a panel of genes believed to be important for recruiting stromal cells to the tumor niches. Treatment of either hMSC or Hs27 cells with TCM from either control or GPC-1 shRNA PC-3 cells did not alter the expression of GPC-1 in either stromal cell culture (Fig. 6A). In contrast, the MMP-9 mRNA expression was increased in both cell types, which correlates to the increase in activity observed in the gelatin zymography assay. In general, hMSC were more responsive to TCM than Hs27 cells as higher levels of mRNA induction were seen in hMSC for the cell migration genes (CXCR4, Rac1, ROCK1-2, Acta2, RhoA, CDC42, and Pak-1), endocrine/paracrine factors (PLA2G2A, SDF1, TGFβ3, and IGF2), and ECM components (FN1, Col5a1 and HAS1). Of note, we observed downregulation of IGF2 in Hs27 cells treated with GPC-1 shRNA PC-3 TCM. There was no pronounced change in the morphology of stromal cells in TCM collected from control and GPC-1 KD PC-3 (Fig. 6B).

**Figure 7:**
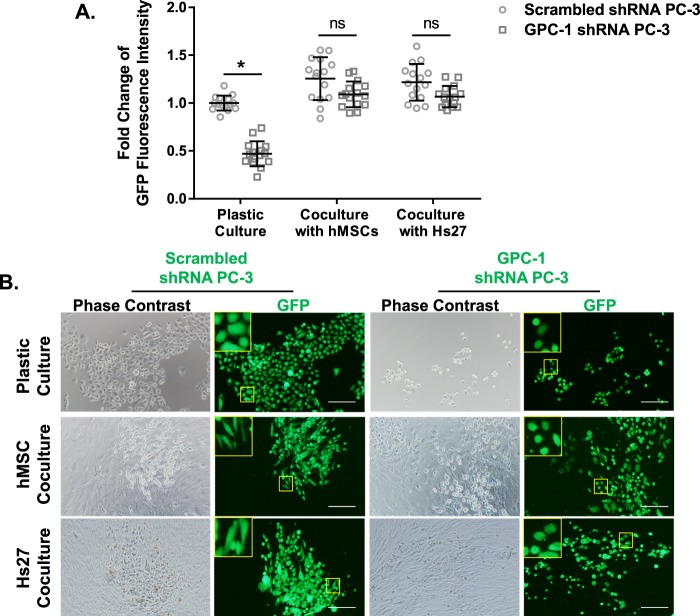
Effect of Stromal Cells on GPC-1 shRNA PC-3 Growth. Scrambled and GPC-1 shRNA PC-3 cells were co-cultured with hMSC and Hs27 at 1:100:100 (cancer cells: hMSC: Hs27) cell ratio and incubated for 7 days. (**A**) Cell growth after 7 days of culture was determined by GFP fluorescent intensity and normalized to control plastic culture. (**B**) The effect of stroma cell on cancer cell morphology was visualized under a GFP filtered fluorescent microscope. The scale bar in **B** indicates 100 µm. Data in **A** are representative of three independent experiments of 5 samples in replicate. *Indicates a significant difference (*p* < 0.05) as compared to control as determined using a two-way ANOVA followed by Dunnett’s post-hoc test; ns indicates difference was not statistically significant.

Furthermore, we co-cultured PC-3 cells with stromal cells to determine the effect of stromal cells on cancer cell growth (Fig. 7A) and morphology (Fig. 7B) after 7 days. Time points beyond this were not investigated as cells tended to detach from the plastic surfaces used after a long-period of culture at confluence, which correlates to reports of enhanced cell contraction, and increased protein deposition from both cancer and stromal cells with this model^58,59^. Inhibition of GPC-1 in PC-3 reduced GFP intensity correlating to lower cell number in single cell culture on plastic surface. This agrees with the cell growth data using crystal violet staining (Fig. 2C,D). In addition, the presence of stromal cells increased cancer cell proliferation. Interestingly, the inhibition of cell growth induced by loss of GPC-1 was rescued in the presence of stromal cells (Fig. 7A). The cell morphology of scrambled shRNA PC-3 cells appeared to be more elongated in the presence of hMSCs or Hs27, as compared to those grown in a single cell culture model (Fig. 7B). However, similar changes in morphology were not detected when GPC-1 expression was inhibited. Those pieces of evidence suggest that the presence of GPC-1 is needed to support changes in gene expression in stromal cells, as well as changes in cell morphology in cancer cells in a co-culture model.

## Discussion

The ability of GPCs to sequester growth factors (eg. FGF2, IGF, VEGEFα and etc.), to mediate cell-extracellular matrix and cell-cell communication, and to trigger cell signaling pathways makes these proteins an attractive nexus for coordinating cell growth and invasion, especially in cancer^2,3,8,10,60^. In support of this hypothesis, studies have suggested roles of select GPCs, such as GPC-3 in the growth of several different cancers, including those derived from the pancreas, breast and the liver^2,3,20,21,61^. In addition, GPC-5 was reported as a tumor suppressor in prostate cancer but was as an oncogene in lung cancer^30,62,63^. These studies suggest that the role of GPCs in cancer is dependent not only on the cancer, but also on the type of GPC isoform expressed in cancer cells.

Compared to other cancers, the role of GPCs in prostate is less studied. Current studies have focused primarily on the differential expression of GPCs in prostate cancer tissue^7,11–13^, and at least GPC-1 and −5 have been suggested to correlate to tumor aggressiveness. Of note, GPC-5 expression was lower in prostate cancer tissue, as compared to adjacent normal controls, which also correlated to decrease survival. In contrast, GPC-1 expression has been reported to be elevated in prostate cancer patients^12,13^. In comparison to studies in patient tissues, less information exists determining the differential expression of GPCs in different prostate cancer cell lines. One recent study even proposed GPC-1 as a biomarker for prostate cancer and demonstrated high expression of GPC-1 in DU-145 cells^13,64^. Despite these studies, there is a gap in the literature that no study yet evaluated the expression of GPC-1, or any other GPCs in PC-3, LNCaP, and PCS-440-010 cells. Thus, to our knowledge, the RT-PCR and immunoblot data reported in this study were among the first to demonstrate the differential expression of these GPC isoforms in these prostate cell lines. These data further showed that the expression of these proteins correlates to the more aggressive prostate cancer cell lines. The current study is limited by the number and type (immortalized human cancer) of cell lines studied, the fact that patient samples usually contain both cancer and stromal cells, and that data on mRNA expression does not always correlate to protein expression between different cell lines^65^.

While few studies exist determining the differential expression of GPCs in prostate cancer cells, even fewer exist determining the function of GPCs in prostate cancer cell growth. We focused on the role of GPC-1 as GPC-1 expression was shown to be increased in more aggressive prostate cancer cells in our own study as well as in prostate cancer tissues^13^. To our knowledge, data reported herein demonstrating that GPC-1 inhibition decreases PC-3 cell growth are among the first to suggest that GPC-1 mediates prostate cancer cell growth. Similar findings have been reported in breast cancer cells^5,8^, and in certain pancreatic cancer cell lines^10,44^. Studies in non-prostate cells have suggested that decreased cell growth may be linked to decreased growth factor signaling, especially those that bind to heparan sulfate^5^. This correlates with the fact that the heparan sulfate chains in GPC-1 can interact with a subset of growth factors that activate tyrosine kinase receptors, as well as the Wnt, and Shh signaling pathways^1^. Thus, it’s possible that decreased PC-3 cells growth is a result of a decrease in receptor activation and expression of certain transcription factors. This hypothesis is supported by our qRT-PCR data demonstrating that inhibition of GPC-1 in PC-3 cells decreased the expression of the chemokine receptor CXCR4, the transcription factor ZEB1 and the epithelial-mesenchymal transition marker, vimentin (VIM). However, inhibition of GPC-1 expression also led to a slight increase in the expression of GPC-5, which can also contribute to cancer progression^30,62,63^. Future studies are needed to determine the exact mechanisms mediating decreased cell growth *in vitro* when GPC-1 is inhibited and whether GPC-5 plays a role in regulating cell growth.

Even though GPC-1 is also detected in DU-145, transiently inhibiting expression of GPC-1 led to a mesenchymal cell morphology correlating to increase cell proliferation and migration. The data suggest that GPC-1 can possibly play a role as a tumor suppressor in DU-145 cells and the role of GPC-1 in mediating cell growth and migration is cell-type dependent. The opposing roles of GPC-1 in these prostate cancer cells may be regulated by other receptors (Sonic Hedgehog^66^ and FGFRs^67^) which can be differentially expressed in these two cell types. In fact, FGFR1 was showed to be highly expressed in DU-145 compared to PC-3 while expression of Sonic Hedgehog is higher in PC-3^68^. The differential expression of these receptors, which co-function with GPC-1 to mediate cell behaviors, may contribute the determination of the role of GPC-1 in these cells. Further evidence of a cell-dependent regulation of GPC can be seen by that fact that inhibition of GPC-1 in DU-145 cells resulted in differential expression of other GPC isoforms, as compared to PC-3 cells (Supplemental Fig. 2). Additional studies will be needed to address this observation. It should be noted that we were unable to establish a stable knockdown of DU-145 cells. This suggest a critical role for GPC-1 in DU-145 cell growth. Unfortunately, this limits our studies *in vivo* to identify the effect of GPC-1 in DU-145 cells on tumor growth *in vivo*.

The demonstration that GPC-1 inhibition enhanced PC-3 tumor growth is in direct contrast to the *in vitro* data. It should be first stated that it is not unusual to see differences on the effect of protein inhibition between *in vitro* and *in vivo* models, especially when it comes to cancer studies^69–72^. In addition, carcinogenesis and metastasis depend not only on the intrinsic gene alterations ongoing inside the cancer cells (i.e. gene expression and mutation), but also on the responsiveness of the stroma^59^. One of the more probable explanations is that the contribution of stromal cells and their TME to GPC-1 signaling is not accounted for using the *in vitro* models employed in this study. This is one reason why we initiated studies assessing the effect of TCM on gene expression and cell morphology. This is also relevant for GPC-1 as this protein coordinates interactions between cells and extracellular signals^8,47,73^. In addition, GPC-1 is known to be a component of exosomes, which are critical for signaling to the TME^74,75^. Thus, it is possible that GPC-1 inhibition may alter growth in single cell cultures due to lack of interaction with growth factors in the media. Such interactions *in vivo* would be supplied by stromal cells, such as fibroblast or stem cells. Regardless of the cause for these differences, the data should serve as cautionary tale with regards to assessing mechanisms of action or potential anti-cancer targets based on *in vitro* data alone.

While these data are among the first to report that inhibition of GPC-1 alters prostate tumor growth *in vivo*, they are not the first to suggest roles for GPC-1 in mediating tumor growth. For example, inhibition of GPC-1 in PANC-1 pancreatic cancer cells decreased the ability of these cells to form tumors in nude mice^10^. Further, there have also been studies assessing the effect of GPC-1 on the growth of breast cancer derived tumors^8,73^. GPC-3 deficiency in mice leads to an overgrowth syndrome, which aligns with its reported function to inhibit of cell proliferation^1,76^. There are also several studies determining the role of GPC-3 on tumor growth^9,17,19–21^. In fact, GPC-3 is suggested to act as an oncogene in hepatocellular carcinoma^1^. In contrast, studies also show that increased GPC-3 expression in breast cancer cells inhibited lung cancer metastasis suggesting a protective role^9^. These studies show that it is not unprecedented that inhibition of GPC-1 could increase tumor growth and that the role of GPCs in cell growth is cancer- and protein-dependent. The differences between the role of GPC-1 in these models may be mediated by differences in the microenvironment and the type of stromal cells present. The level of vascularization may also be important and should be addressed in future studies.

As stated above, the mechanisms mediating the increase in tumor growth in mice injected with GPC-1 shRNA PC-3 cells are not fully known. However, data from this current study suggest possible roles for MMP-9 and N-Cad expression in that changes in the expression of these proteins correlated to changes in cell and tumor growth. Increased MMP-9 expression is known to correlate to more advanced prostate cancer in human patients^77–79^. Increase in MMP-9, as well as other MMPs, are believed to facilitate cancer dissemination and interactions with stroma cells^77^ by remodeling the extracellular matrix^80^ and activating integrin subunits^77,81,82^. Interestingly, the expression of intrinsic MMP-9 mRNA in PC-3 cells was not altered in PC-3 cells when GPC-1 was inhibited. Thus, the increase in MMP-9 expression in tumors may be mediated by changes in interactions with stromal cells in the TME. Our data supports this hypothesis by demonstrating the increase in MMP-9 expression and activity in hMSC and Hs27 treated with TCM from GPC-1 shRNA PC-3 cells. While no studies could be found identifying direct interactions between MMP-9 with GPC-1, it has been reported that MMP-9 associates with GPC-like proteoglycans in mouse colon adenocarcinomas^83^. GPC-1 and MMP-9 expression have also been correlated in pancreatic cancer^84^. The mechanisms of how GPC-1 inhibition increases MMP-9 activity is under study.

A striking dichotomy between data reported in this study and the literature is that GPC-1 expression is increased in prostate tumors and correlates to aggressiveness^7,13^. This would suggest that GPC-1 is an oncogene, a hypothesis supported by our observation of a reduction in cell growth in a single cell culture model. However, it should be emphasized that because a protein is overexpressed in a tumor does not mean it’s a driver of carcinogenesis. Rather, the overexpression of GPC-1 may be a secondary event subsequent to transformation. In the models presented in this study GPC-1 may act as a tumor suppressor in the presence of stromal cells. Evidence showed in the TCM studies (Fig. 6A) suggested that GPC-1 expression in cancer cells is involved in recruitment of specific cells (eg. hMSC and fibroblasts) to the TME to support cancer growth by upregulating migratory and chemokine-tracking factors. These factors have been showed to be involved in chemotaxis (CXCR4^85^) and locomotion (ROCK1/2, Acta2, Pak-1, Rac1, and RhoA^28^) of stromal cells to help migration of these cells to tumor niches. At the same time, these stromal cells secrete soluble factors and increase collagen and fibronectin to support cancer cell growth and transformation.

To determine the correlation between GPC-1 expression and prostate cancer in humans we assessed the TCGA Prostate Cancer Cohort in which samples were collected at the primary tumor sites and only mRNA expression data are available. Interestingly, we observed a reduction of GPC-1 mRNA within the primary tumor population (Supplemental Fig. 9A). Patients with low GPC-1 expression also had lower survival rate after 7 years of diagnosis (Supplemental Fig. 9B). These data are in agreement with recent reports of GPC-1 reduction in serum, plasma, and urine from prostate cancer patients^13,86^. Collectively, it is likely that GPC-1 may play a protective role in prostate cancer. However, future studies are urgently needed because the TCGA Prostate Cancer Cohort doesn’t truly reflect the prognosis of prostate cancer patients, especially those who acquire bone or brain metastasis. In addition, samples reported in Shore *et al*.^86^ were presumably circulating GPC-1, which were cleaved by proteases^87^ and the expression of proteases have not been reported elsewhere. Future studies also need to focus on these factors as well because fragmented GPC-1 may have pharmacological effects to specific cell types.

In conclusion, our findings demonstrate that molecular inhibition of GPC-1 has paradoxical effects on prostate cancer cell and tumor growth. *In vitro*, GPC-1 appears to act as an oncogene and mediate cell growth and migration. The role of GPC-1 in cell growth appears to be cell-type dependent. In contrast, *in vivo* GPC-1 appears to act as tumor suppressor, possibly by mediating extracellular signaling and cancer cell interaction with the TME. The differential effects of TCM isolated from GPC-1 inhibited cells on tumor stromal cell gene expression further support the hypothesis that GPC-1’s role in prostate cancer includes mediating interactions with the TME.

## Supplementary information

Supplemental Data

## Data Availability

The datasets generated during and/or analyzed during the current study are available from the corresponding author on reasonable request.
