# Supplementary material for: Paradoxical Role of Glypican-1 in Prostate Cancer Cell and Tumor Growth
Source: Sci Rep. 2019 Aug 7;9:11478. doi: 10.1038/s41598-019-47874-2 (PMC6685992; doi:10.1038/s41598-019-47874-2)
Supplement: Supplementary file 1 — Supplemental Data [file 41598_2019_47874_MOESM1_ESM.pdf]

Paradoxical Role of Glypican-1 in Prostate Cancer Cell and Tumor Growth

Nhat D. Quach[1,5], Sukhneeraj Pal Kaur[1], Matthew Eggert[2], Lishann Ingram[1], Deepraj Ghosh[5], Sheela Sheth[4], Tamas Nagy[3],

Michelle R. Dawson[5,6,7], Robert D. Arnold[2,8], Brian S. Cummings[1,8,*].

[1]*Department of Pharmaceutical and Biomedical Sciences, College of Pharmacy, University of Georgia, Athens, GA, USA*

[2]*Department of Drug Discovery & Development, Auburn University, Auburn, AL, USA*

[3]*Department of Pathology, College of Veterinary Medicine, University of Georgia, Athens, GA, US*

[4]*Medical College of Georgia, Augusta University, Augusta, GA, USA*

[5]*Department of Molecular Pharmacology, Physiology, & Biotechnology, Brown University, Providence, RI, USA*

[6]*Center for Biomedical Engineering, Brown University, Providence, RI, USA.*

[7]*School of Engineering, Brown University, Providence, RI, USA*

[8]*Interdisciplinary Toxicology Program, University of Georgia, Athens, GA, USA*

*Corresponding Author

336 College of Pharmacy South
University of Georgia
Athens, GA 30607
Phone: 706-542-3792
Fax: 706-542-5358
E-Mail: briansc@uga.edu

Key words: Glypican-1**,** prostate cancer**,** tumor microenvironment, prostate, bone marrow-derived mesenchymal stem cells, fibroblasts.

Supplemental Figure 1: Effect of GPC-1 Inhibition on GPC-1 Expression and Cell Growth and Morphology in DU-145 Cells. A. DU-145 cells were transfected with GPC-1 shRNA (Genecopoeia, Rockville, MD) prior to analysis of GPC-1 expression using immunoblot analysis. Effect of GPC-1 inhibition on DU-145 cells morphology (**B**), proliferation (**C**) and migration (**D**) were assessed using phase contrast microscopy, crystal violet staining (11 days) and scratch assays, respectively. Data in **C** and **D** are presented as the mean ± the S.E.M. of at least 3 (n = 3) separate passages. *Indicates a significant difference ($p < 0.05$) as compared to control as determined using a Student t-test.

Supplemental Figure 2: Effect of GPC-1 Inhibition on the Expression of GPC Isoforms. qRT-PCR was used to measure the mRNA expression level of GPC isoforms in PC-3 (**A**) and DU-145 (**B**) cells transfected with GPC-1 shRNA (Sigma, St. Louis, MO). Data are presented as the mean ± the S.E.M. of at least 3 (n = 3) separate passages. *Indicates a significant difference ($p < 0.05$) as compared to control as determined using a Student t-test.

Supplemental Figure 3: Effect of GPC-1 Inhibition Using Different shRNA Plasmid Sequences on Prostate Cancer Cell Growth and Morphology. A. Effect of GPC-1 shRNAs (Sigma, St. Louis, MO) on expression of GPC-1 in PC-3 cells. **B.** Effect of GPC-1 inhibition on cell morphology visualized by crystal violet staining at low density. **C.** Changes in cell growth in GPC-1 knockdown PC-3 cells visualized by crystal violet staining after 11 days of culture. **D.** Inhibition of GPC-1 results in decrease in cell migration in the scratch wound assay as visualized by crystal violet staining. Data in **C** and **D** are representative of at least 3 separate experiments with at least 3 independent cell passages. Data in **C** and **D** are presented as the mean ± the S.E.M. of at least 3 (n = 3) separate passages. *Indicates a significant difference ($p < 0.05$) as compared to control as determined using a Student t-test.

Supplemental Figure 4: H&E Staining of Tumor Section from Scrambled (A) and GPC-1 shRNA (B) Mouse xenografts. Data in **A** and **B** are representative of at least three individual mice. The scale bar indicates 50 µm.

Supplemental Figure 5: Ki-67 Expression in Tumor Sections from Scrambled (A) and GPC-1 shRNA (B) Mouse Xenografts. Data in **A** and **B** are representative of at least 3 separate mice. Data in **C** are presented as the mean ± S.E.M. of at least 5 separate images taken from at 5 different section of each tumor slice. The scale bar in **A** and **B** indicates 50 µm.

Supplemental Figure 6: E-Cad and N-Cad Staining in Tumor Sections from Scrambled (A) and GPC-1 shRNA (B) Mouse Xenografts. Data in **A** and **B** are representative of at least 3 individual mice. E-Cad staining is indicated by red fluorescence while N-Cad is indicated by green. The scale bar indicates 50 µm.

Supplemental Figure 7: Effect of GPC-1 Inhibition on MMP9/2 Activity. TCM from control PC-3 cells (scrambled shRNA) and from GPC-1 shRNA cells were collected and applied to confluent Hs27 and hMSCs at confluence for 24 hours. Media from these stromal cells were collected for MMP-9 and 2 activity analysis using gelatin zymography.

Supplemental Figure 8: GPC-1 mRNA Expression Profile in Human Prostate Cancer Patients and Correlation to Prostate Cancer Patient Survival. A TCGA Prostate Cancer (PRAD) cohort of 550 patients was analyzed using the UCSC Xena Cancer Database (http://xena.ucsc.edu/). Gene expression RNAseq (ployA+ IlluminaHiSeq pancan normalized) was selected to analyze GPC-1 expression in solid normal and primary prostate tissue samples (**A**). Kaplan-Meier Survival Curves were generated for these same patients (**B**) using the default Xena database algorithm. Even though the statistical log-ranked test (p= 0.9387) does not indicate significant correlation between patient with high and low GPC-1 expression, patients with low expression of GPC-1 (blue lines) had a lower survival rate compared to high GPC-1 expressing patients (highlighted region).

Supplemental Figure 9: Full western blot images used in Figure 1 and 2

Supplemental Figure 10: Full western blot images used in Figure 5

Supplemental Figure 11: Full western blot images used in Supplementary Figure 1 and 2.

Supplemental Figure 1

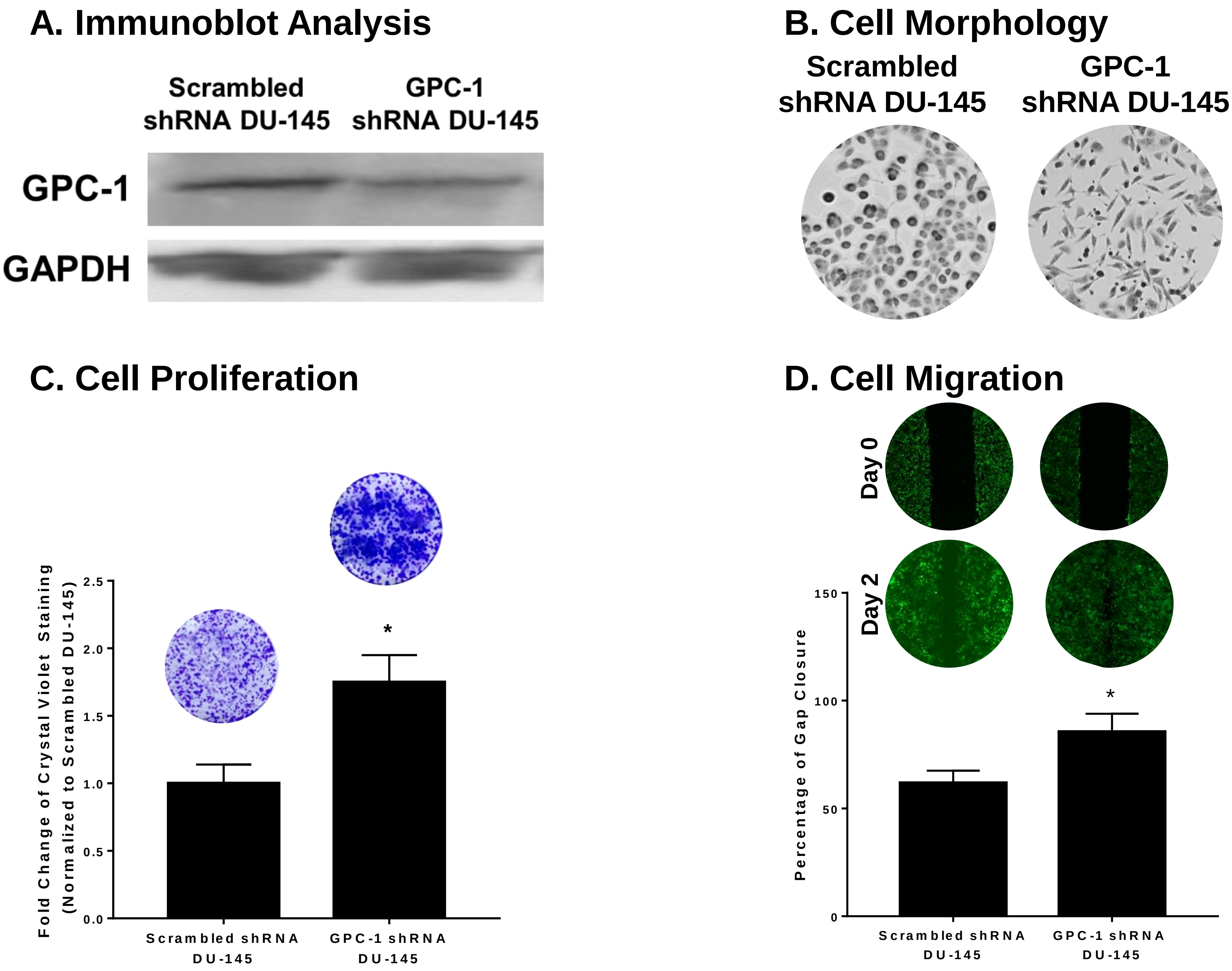

Supplemental Figure 2

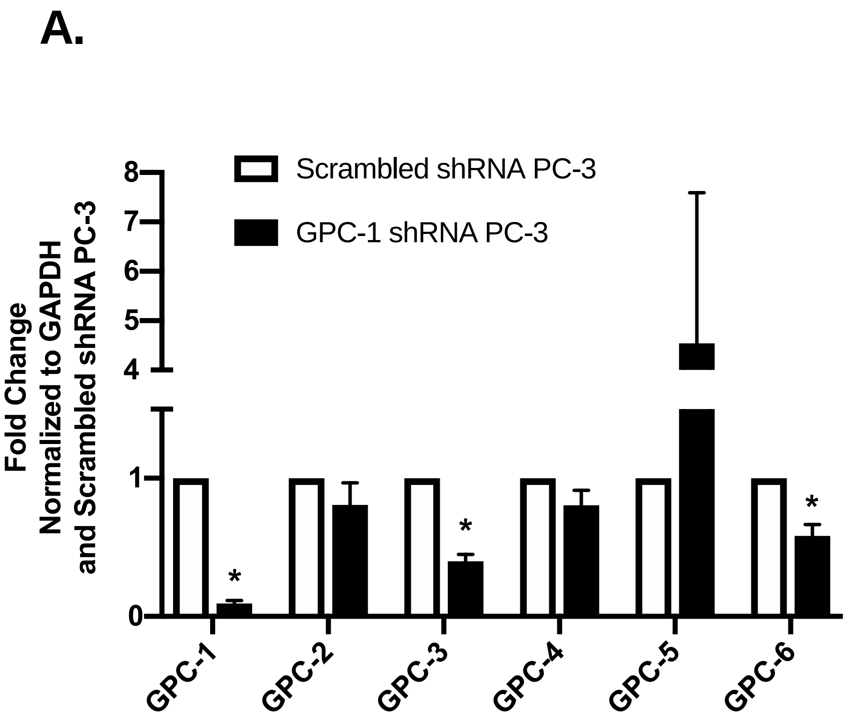

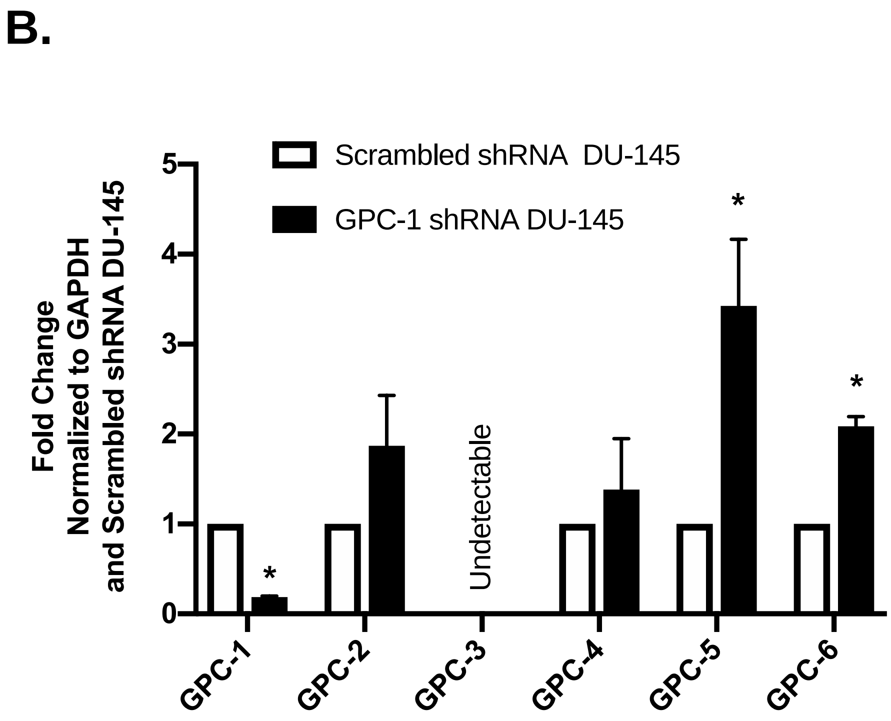

Supplemental Figure 3

A. Immunoblot Analysis

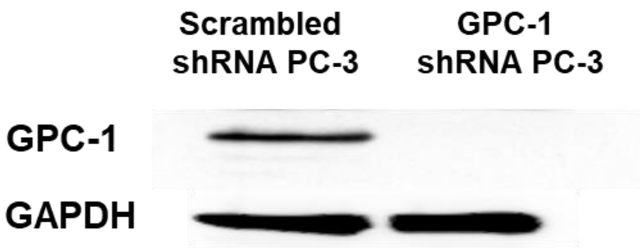

B. Cell Morphology

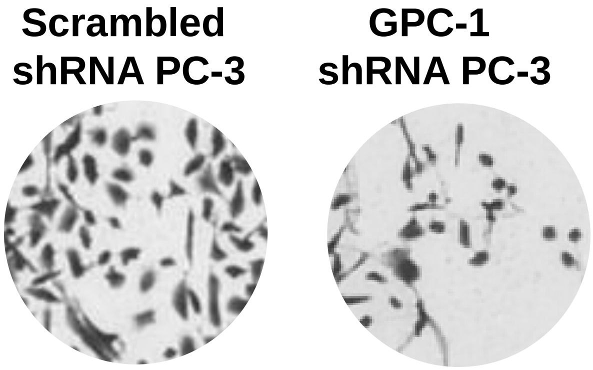

C. Cell Proliferation

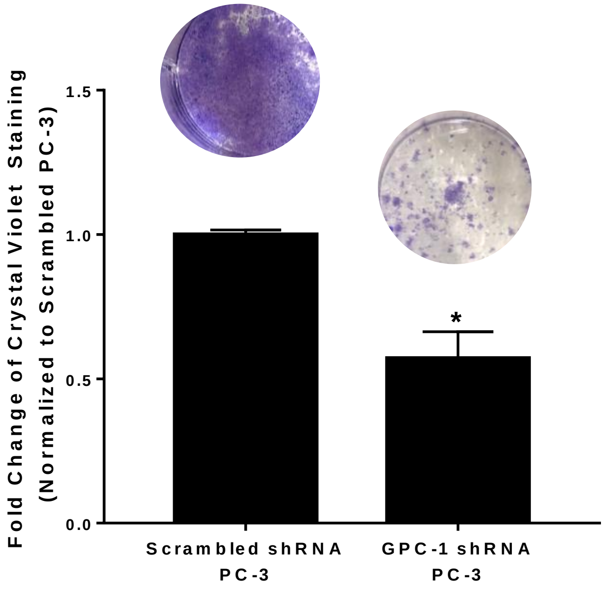

D. Cell Migration

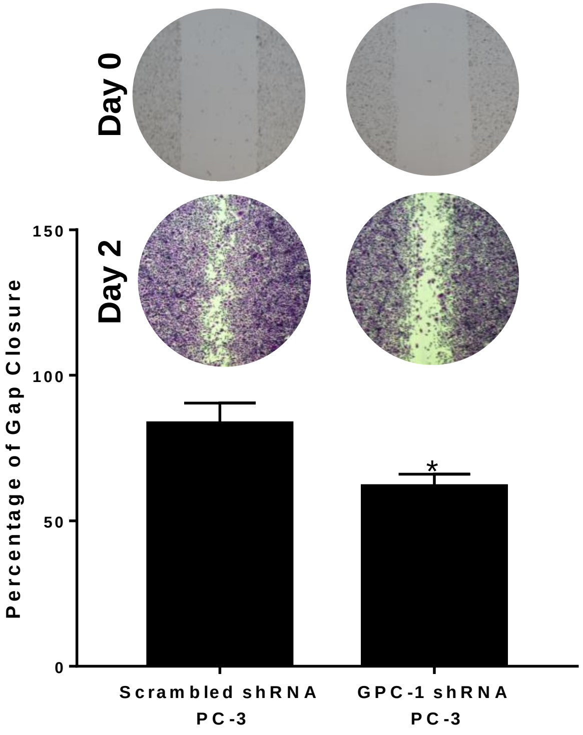

Supplemental Figure 4

A. Scrambled shRNA PC-3

B. GPC-1 shRNA PC-3

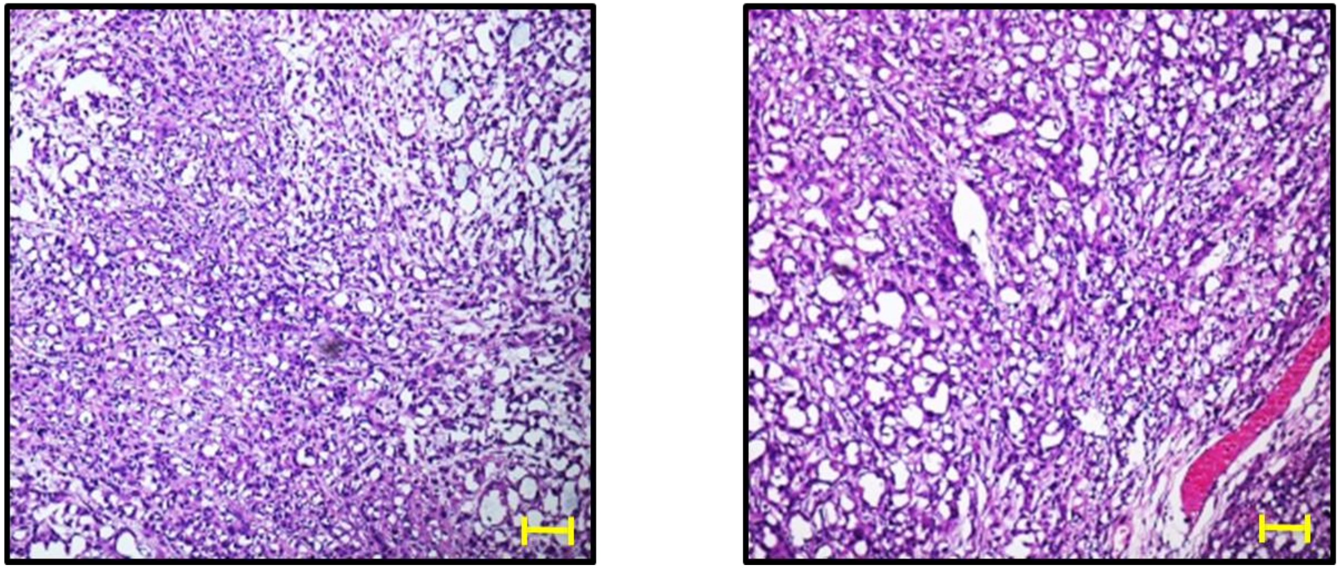

Supplemental Figure 5

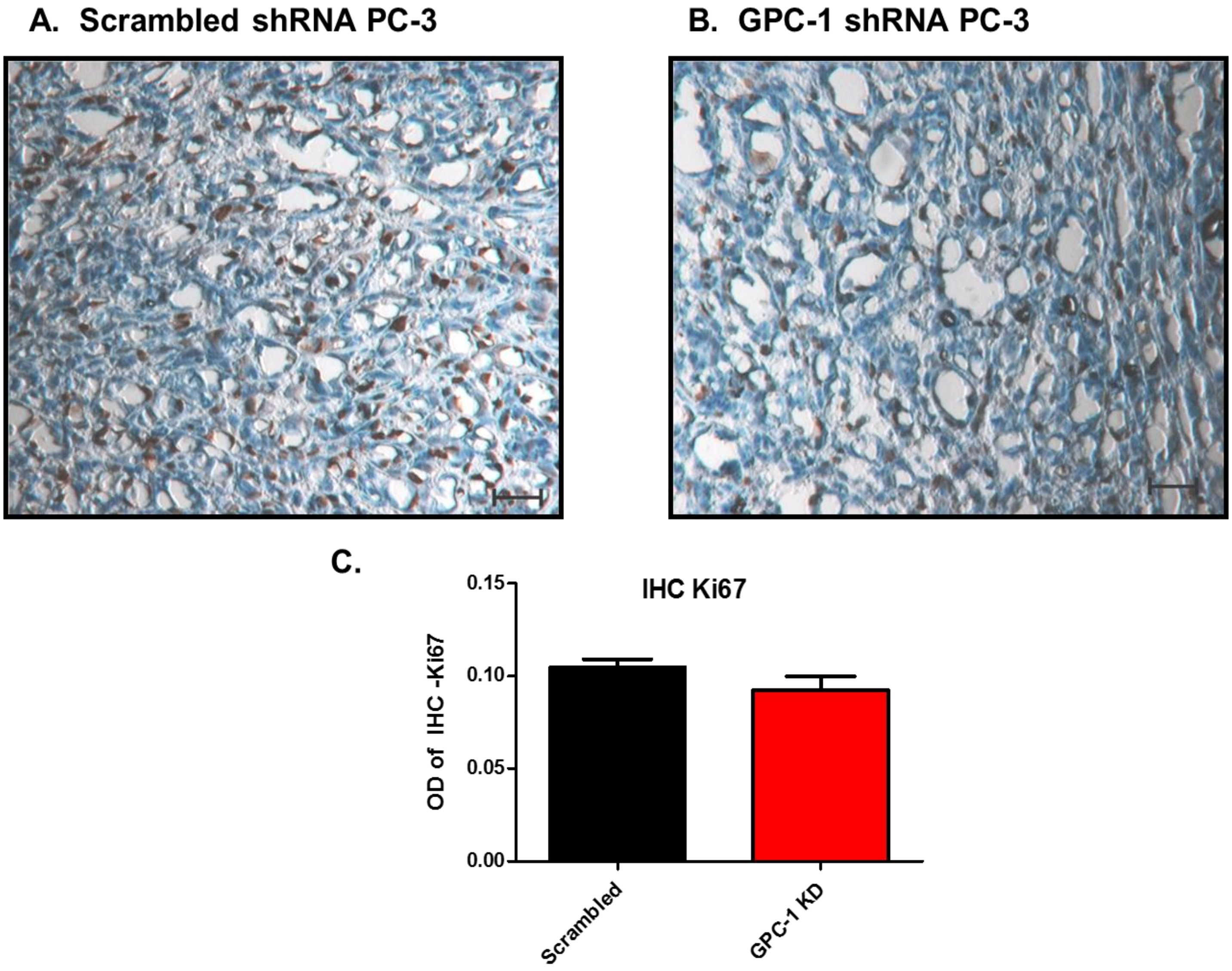

Supplemental Figure 6

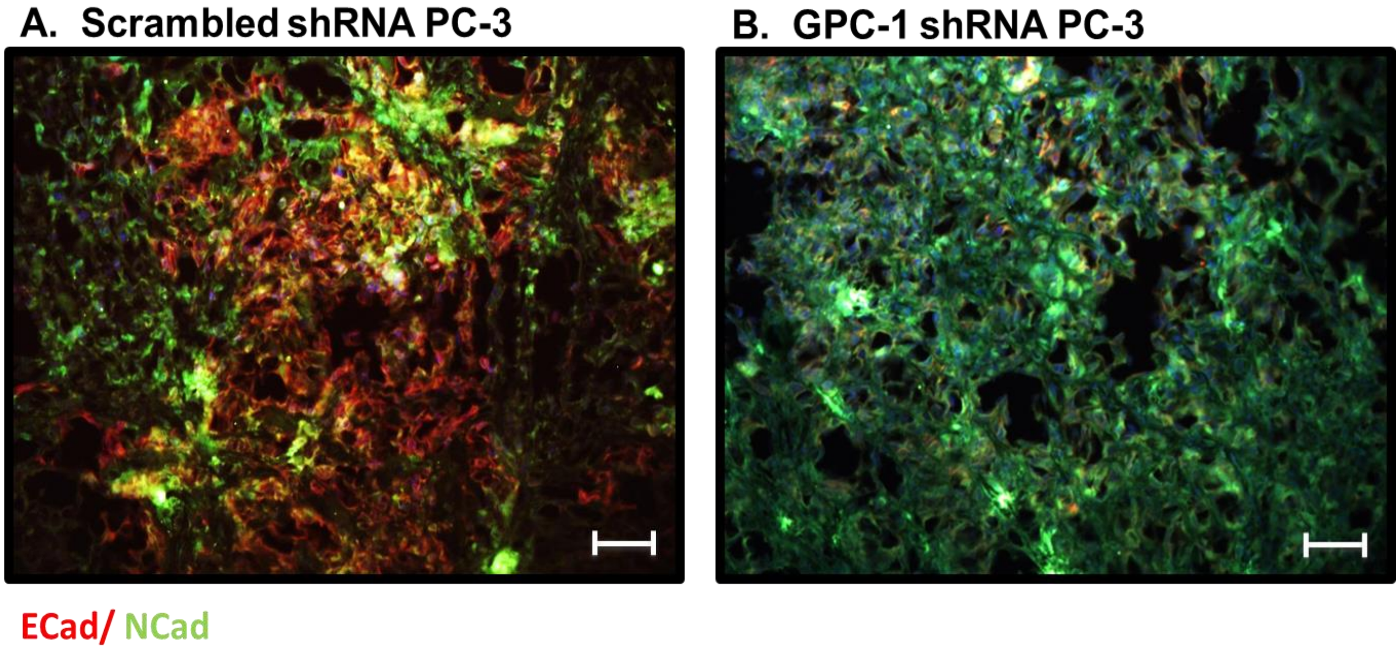

Supplemental Figure 7

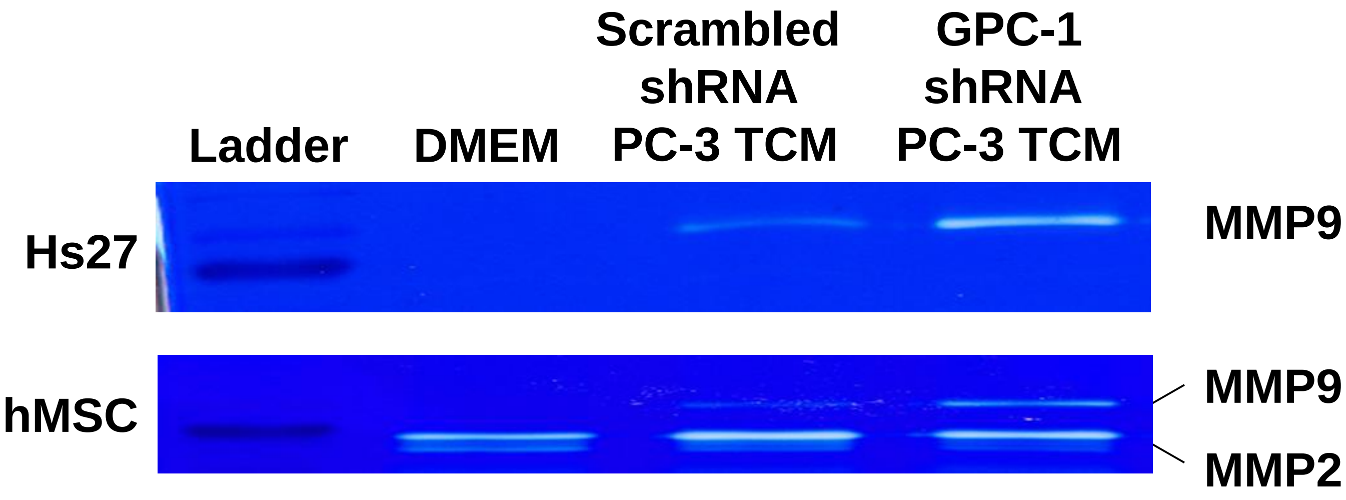

Supplemental Figure 8

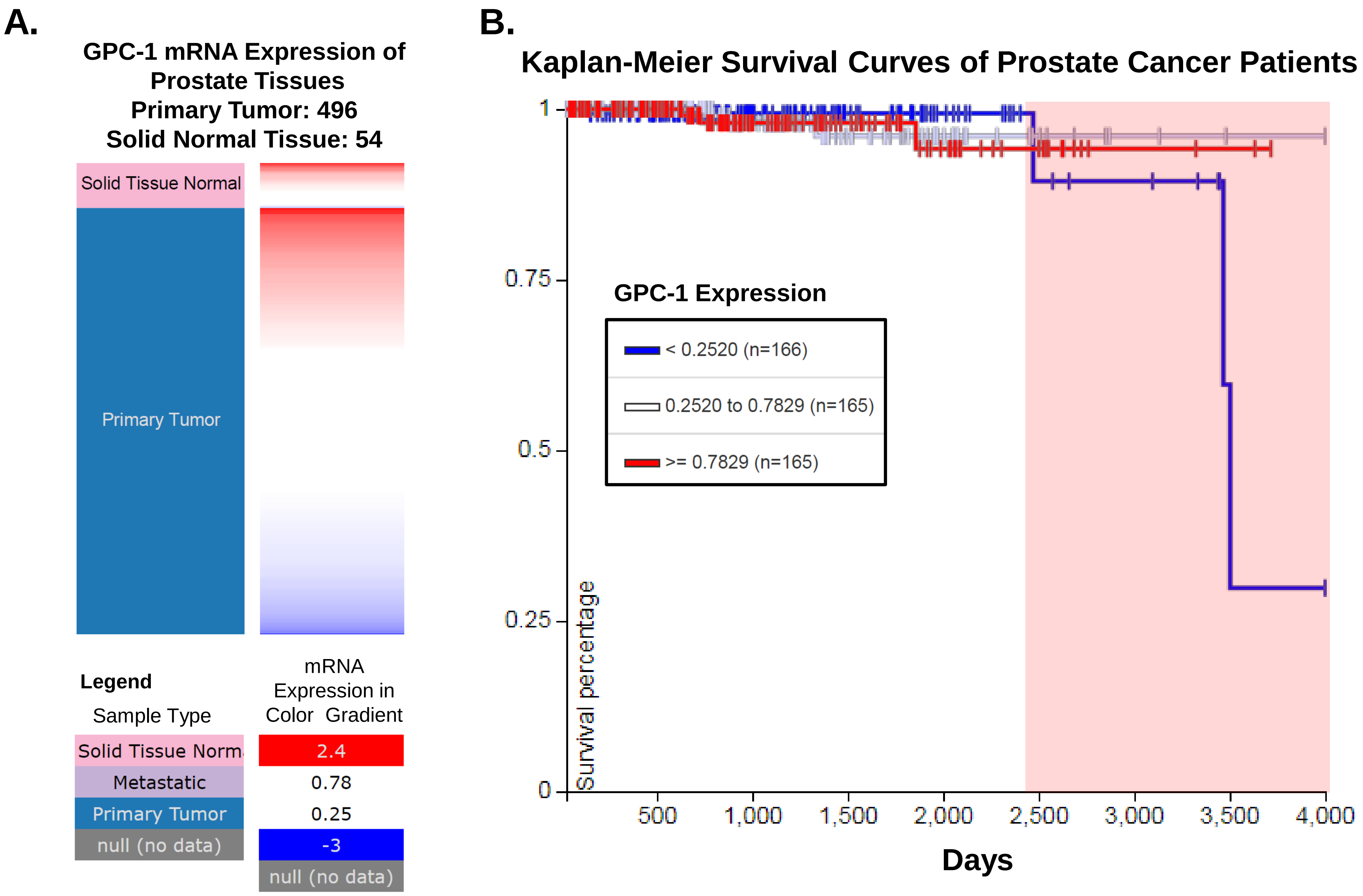

Supplemental Figure 9

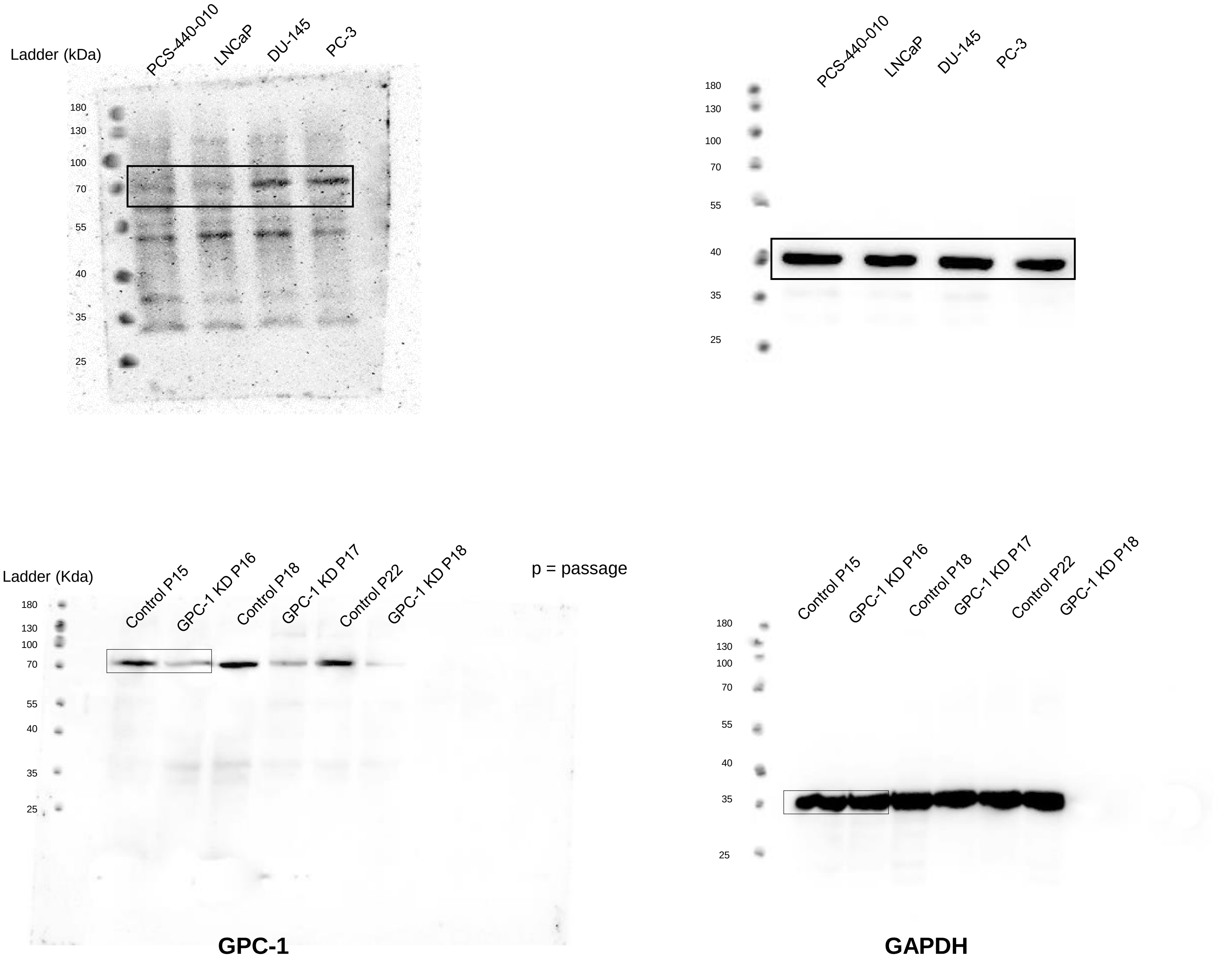

Supplemental Figure 10

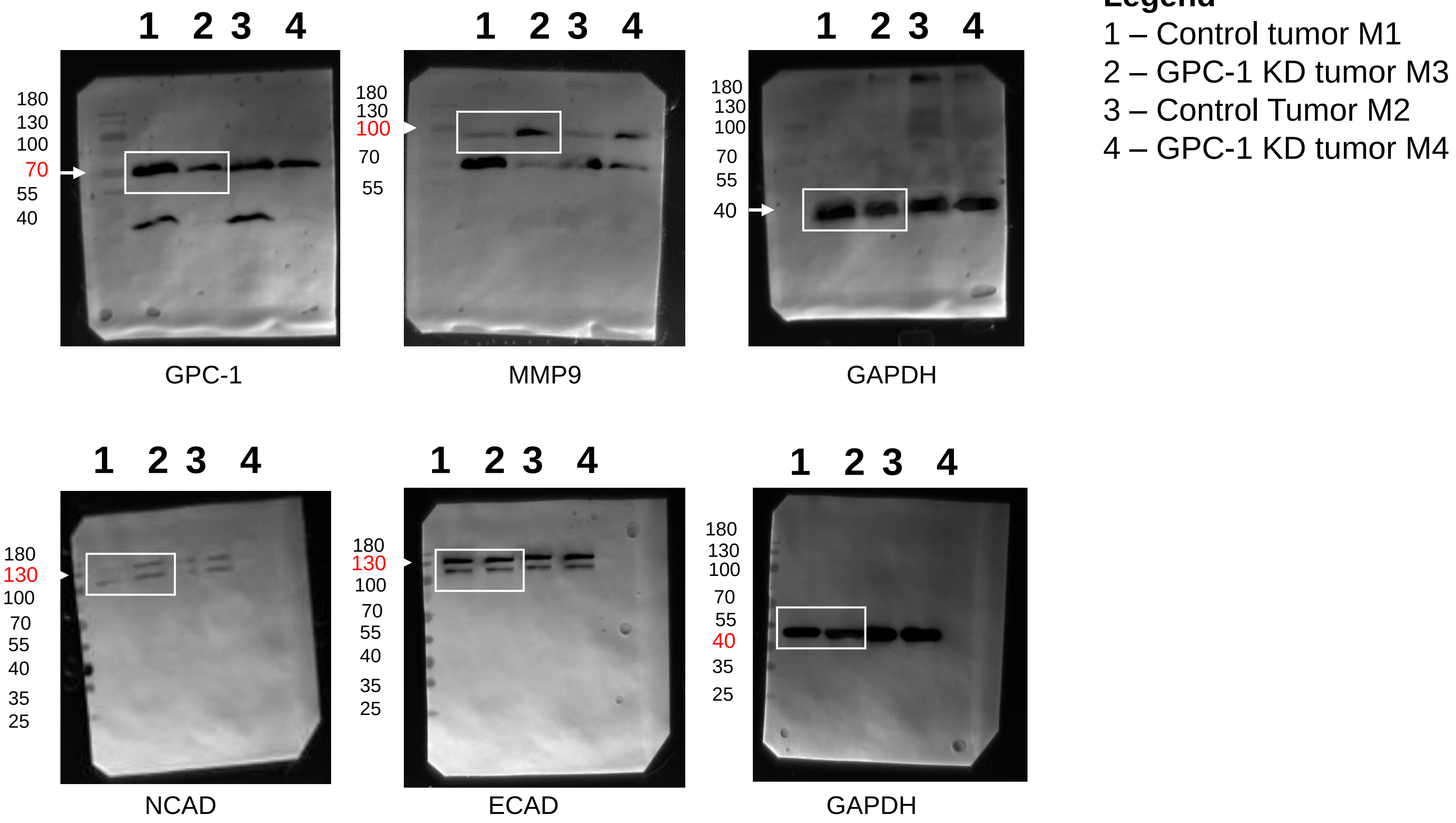

Supplemental Figure 11

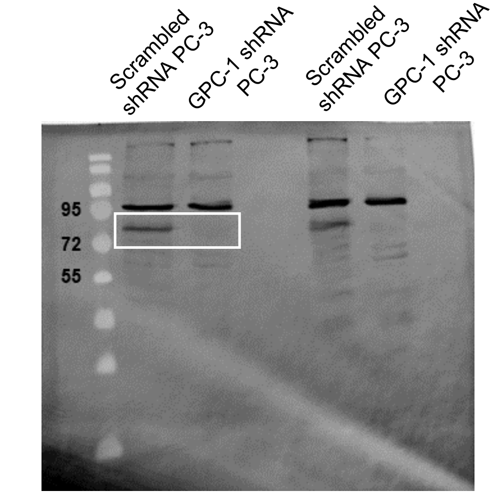

GPC-1

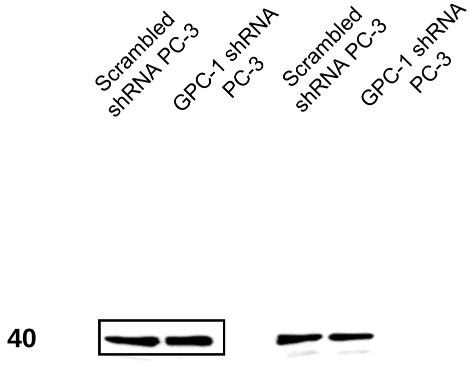

GAPDH

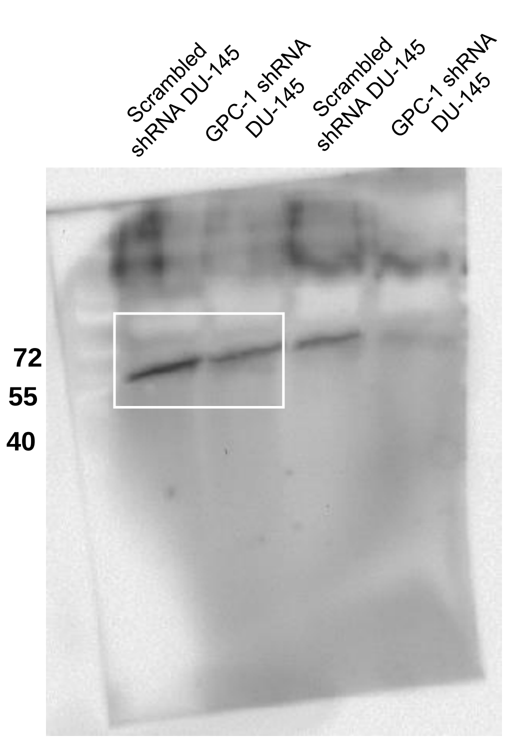

GPC-1

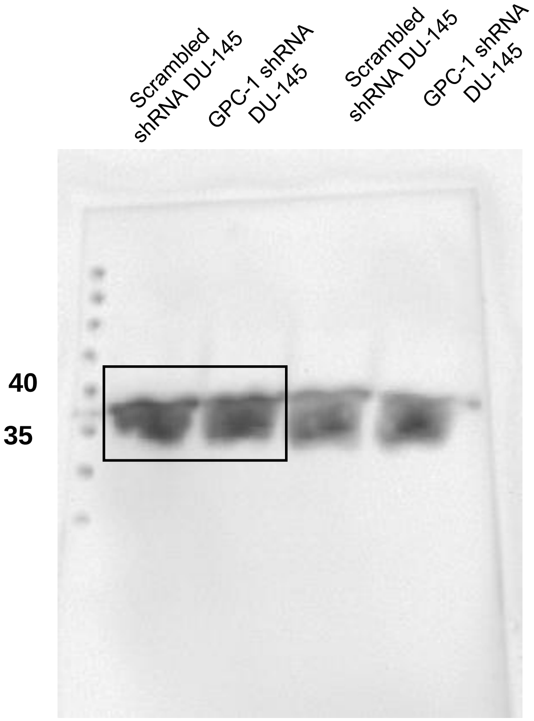

GAPDH
